# ONAC066, A Stress-Responsive NAC Transcription Activator, Positively Contributes to Rice Immunity Against *Magnaprothe oryzae* Through Modulating Expression of *OsWRKY62* and Three Cytochrome P450 Genes

**DOI:** 10.3389/fpls.2021.749186

**Published:** 2021-09-09

**Authors:** Xi Yuan, Hui Wang, Yan Bi, Yuqing Yan, Yizhou Gao, Xiaohui Xiong, Jiajing Wang, Dayong Li, Fengming Song

**Affiliations:** ^1^National Key Laboratory for Rice Biology, Institute of Biotechnology, Zhejiang University, Hangzhou, China; ^2^College of Chemistry and Life Sciences, Zhejiang Normal University, Jinhua, China

**Keywords:** NAC (NAM, ATAF, and CUC), ONAC066, rice immunity, *OsWRKY62*, cytochrome P450s

## Abstract

NAC transcriptional factors constitute a large family in rice and some of them have been demonstrated to play crucial roles in rice immunity. The present study investigated the function and mechanism of *ONAC066* in rice immunity. ONAC066 shows transcription activator activity that depends on its C-terminal region in rice cells. *ONAC066*-OE plants exhibited enhanced resistance while *ONAC066*-Ri and *onac066-1* plants showed attenuated resistance to *Magnaporthe oryzae*. A total of 81 genes were found to be up-regulated in *ONAC066*-OE plants, and 26 of them were predicted to be induced by *M. oryzae*. Four *OsWRKY* genes, including *OsWRKY45* and *OsWRKY62*, were up-regulated in *ONAC066*-OE plants but down-regulated in *ONAC066*-Ri plants. ONAC066 bound to NAC core-binding site in *OsWRKY62* promoter and activated *OsWRKY62* expression, indicating that *OsWRKY62* is a ONAC066 target. A set of cytochrome P450 genes were found to be co-expressed with *ONAC066* and 5 of them were up-regulated in *ONAC066*-OE plants but down-regulated in *ONAC066*-Ri plants. ONAC066 bound to promoters of cytochrome P450 genes *LOC_Os02g30110*, *LOC_Os06g37300*, and *LOC_Os02g36150* and activated their transcription, indicating that these three cytochrome P450 genes are ONAC066 targets. These results suggest that ONAC066, as a transcription activator, positively contributes to rice immunity through modulating the expression of *OsWRKY62* and a set of cytochrome P450 genes to activate defense response.

## Introduction

During their lifespan, plants are always attacked by numerous potential pathogenic microbes including fungi, bacteria and viruses. To survive, plants have evolved to possess a sophisticated innate immune system, which includes two layered immune responses, known as pathogen/microbe/damage-associated molecular pattern (PAMP/MAMP/DAMP)-triggered immunity (PTI) and effector-triggered immunity (ETI) (Jones and Dangl, 2006; Boller and He, 2009; Dangl et al., 2013; Zhang et al., 2020). Upon perception and recognition of pathogen-derived signals, plants often effectively initiate a complicated signaling network (Peng et al., 2018; Zhou and Zhang, 2020), leading to a large-scale transcriptional reprogramming of gene expression in timely and coordinately manners (Tsuda and Somssich, 2015; Li et al., 2016; Birkenbihl et al., 2017; Chen et al., 2020). Transcriptional reprogramming in activation of immune response requires concerted and fine-tuned temporal and spatial functions of numerous transcription factors (TFs) belonging to different families (Buscaill and Rivas, 2014; Birkenbihl et al., 2017; Ng et al., 2018). During the last two decades, extensive genetic and biochemical studies have revealed the importance of TFs belonging to families of WRKY, AP2/ERF, NAC (NAM, ATAF1/2, and CUC2), MYB, bZIP, homeodomain, bHLH, NF-Y, and CAMTA in plant immunity (Nuruzzaman et al., 2013; Buscaill and Rivas, 2014; Dey and Vlot, 2015; Huang et al., 2016; Noman et al., 2017; Ng et al., 2018; Yuan et al., 2019a; Bian et al., 2020; Wani et al., 2021).

NAC family is plant-specific and represents one of the largest plant TF families. NAC TFs are characterized by the presence of highly conserved NAC domains at N-terminal, which determine DNA binding activity, and of variable domains at C-terminal, which are responsible for transcription activity (Olsen et al., 2005). Most NAC TFs exhibit binding activities to NAC recognition sequence (NACRS) *cis*-element with the sequence of CATGT or CACG (Tran et al., 2004), which are frequently present in promoters of certain defense genes. Functional studies using knockout/knockdown mutants and/or overexpression transgenic lines have demonstrated that NAC TFs play significant roles in plant growth, development, and response to biotic and abiotic stress (Puranik et al., 2012; Shao et al., 2015; Kim et al., 2016; Yuan et al., 2019a; Diao et al., 2020; Forlani et al., 2021; Kou et al., 2021).

Genome-wide transcriptome analysis revealed that a set of TF genes including NAC genes is activated by infection of *Magnaporthe oryzae*, the causal agent of blast disease (Kawahara et al., 2012; Sun et al., 2015). A total of 151 members in rice NAC TF family have been identified (Ooka et al., 2003; Fang et al., 2008; Nuruzzaman et al., 2010) and some of the rice NAC TFs have been shown to be involved in rice immunity, acting as either positive or negative regulators. OsNAC6, belonging to ATAF subfamily (Yuan et al., 2019a), positively regulates resistance to *M. grisea* and two genes for a cationic peroxidase (*LOC_Os01g73200*) and a protein containing a conserved DUF26 domain (*LOC_Os04g25060*) were identified as putative target genes of OsNAC6 (Nakashima et al., 2007). *OsNAC111*-overexpressing plants showed increased resistance to *M. oryzae* and constitutively expressed several defense genes, suggesting that OsNAC111, a member of TERN subfamily (Yuan et al., 2019a), positively regulates the promoter activity of a specific set of defense genes including *PR2* and *PR8* and contributes to rice immunity (Yokotani et al., 2014). Mutation in *OsNAC60*, whose transcript abundance is regulated by *miR164a*, and silencing of *ONAC122* or *ONAC131*, coding for NAC TFs belonging to NAP subfamily (Yuan et al., 2019a), increased susceptibility to *M. oryzae* (Sun et al., 2013; Wang et al., 2018), while overexpression of *OsNAC58* increased resistance to *Xanthomonas oryzae* pv. *oryzae*, the causal agent of bacterial blight disease (Park et al., 2017). *ONAC066*, which was induced by the blast fungus, positively regulates resistance to *M. oryzae* and *X. oryzae* pv. *oryzae* through modulating of abscisic acid (ABA) signaling pathway (Liu et al., 2018). RIM1, belonging to the NTL subfamily (Yuan et al., 2019a), negatively regulates rice immunity to Rice dwarf virus by acting as a host factor that is required for multiplication of the virus in host plants and functions as a molecular link in jasmonic acid signaling (Yoshii et al., 2009, 2010). *OsNAC4* was found to be up-regulated during non-host defense response and regulate the occurrence of hypersensitive cell death, accompanied by loss of plasma membrane integrity, nuclear DNA fragmentation and typical morphological changes (Kaneda et al., 2009). Therefore, the NAC TFs play critical roles in different aspects of rice immunity; however, the molecular mechanism for these NAC TFs in rice immunity remains largely unknown.

We previously identified *ONAC066* (*LOC_Os03g56580*) as one of the *M. oryzae*-responsive NAC TF genes (Sun et al., 2015). ONAC066 is classified into the ONAC022 subgroup of group I in NAC TF family (Ooka et al., 2003) and its Arabidopsis homologous ANAC042/AtJUB1 has been shown to be involved in pathogen-induced defense response (Wu et al., 2012; Shahnejat-Bushehri et al., 2016). *ONAC066* has been shown to function as a positive regulator of drought and oxidative stress tolerance as well as disease resistance against *M. oryzae* and *X. oryzae* pv. *oryzae* (Liu et al., 2018; Yuan et al., 2019b). The present study aimed to elucidate the function of *ONAC066* in rice immunity and to identify the putative targets that are regulated by ONAC066. Our data demonstrated that *ONAC066* acts as a positive regulator of rice immunity against *M. oryzae* through modulating expression of *OsWRKY62*, a WRKY TF that is involved in rice immunity (Fukushima et al., 2016; Liu et al., 2016), and three cytochrome P450 genes.

## Materials and Methods

### Plant Materials and Growth Conditions

*ONAC066*-OE and *ONAC066*-RNAi transgenic lines in background of rice (*Oryza sativa* L.) subsp. *japonica* cv. Zhonghua 11 (ZH11) were identified as previously described (Yuan et al., 2019b). Seeds of T-DNA insertion mutant *onac066-1* (1C-13513) in *japonica* cv. Hwayoung (HY) background were obtained from POSTECH RISD (Rice T-DNA Insertion Sequence Database) (Jeon et al., 2000; An et al., 2003). Homozygous mutants for T-DNA insertion were identified by genomic DNA PCR with gene-specific and T-DNA-specific (2707RB) primers (Supplementary Table S1). Rice plants were grown in a growth room with a cycle of 14 h of light (28°C) and 10 h of darkness (24°C), as previously described (Hong et al., 2017).

### Disease Assays

*Magnaprothe oryzae* isolate RB22 was grown on complete medium [10 g glucose, 2 g peptone, 1 g yeast extract, 1 g casamino acids, 0.l% (vol/vol) 1000× trace elements (2.2 g ZnSO_4_∙7H_2_O, 1.1 g H_3_BO_3_, 0.5 g MnCl_2_∙4H_2_O, 0.5 g FeSO_4_∙7H_2_O, 0.16 g CoCl_2_∙6H_2_O, 0.15 g NaMoO_4_∙5H_2_O, 5 g NaEDTA, 100 mL ddH_2_O), 0.l% (v/v) 1000× vitamin supplement (10 mg biotin, 10 mg pyridoxin, 10 mg thiamine, 10 mg riboflavin, 10 mg *p*-aminobenzonic acid, 10 mg nicotinic acid, 100 mL ddH_2_O), 6 g NaNO_3_, 0.5 g KCl, 0.5 g MgSO_4_, 1.5 g KH_2_PO_4_, pH6.5, 1L] (Talbot et al., 1993) at 25°C for 10 days and spores were collected to prepare spore suspension inoculum (5 × 10^5^ spores/ml containing 0.02% Tween-20). For whole plant inoculation assays, 4-week-old plants were inoculated by foliar spraying with spore suspension inoculum, as described previously (Hong et al., 2016). For detached leaf inoculation assays, fully expanded leaves from 4-week-old plants were inoculated by dropping 5 ml spore suspension inoculum on leaf surface. The inoculated plants and leaves were kept in dark for 24 h at 25°C with 100% relative humidity and then moved to the growth room with normal growth condition. Disease phenotypes were evaluated and samples were collected at 5 days post inoculation (dpi). Fungal growth in inoculated leaves was quantified by measuring *M. oryzae* genomic DNA relative to rice genomic DNA by qPCR (Zellerhoff et al., 2006). Total genomic DNA was extracted from leaves in extraction buffer containing 2% (wt/vol) CTAB, 20 mM EDTA, 1.4 M NaCl, and 100 mM Tris-HCl (pH8.0), and purified with chloroform/isoamyl alcohol (24:1, vol/vol), followed by isopropyl alcohol precipitation. Genomic DNA of *M. oryzae* and rice was determined using specific primers for *M. oryzae MoPot2* DNA and rice *OsUbi* DNA, respectively, by qPCR, as previously described (Li et al., 2017). The primers used were listed in Supplementary Table S1. Relative fungal growth was presented as ratios obtained by comparison of the fungal *MoPot2* genomic level with rice *OsUbi* genomic DNA level.

### RNA Isolation and qRT-PCR

Total RNA was extracted using RNA Isolater reagent (Vazyme, Nanjing, China) and treated with genome DNA wiper mix (Vazyme, Nanjing, China) at 42°C for 2 min to remove the remaining genomic DNA. First-strand cDNA was synthesized with 2 μg purified total RNA using the reverse transcription system (Vazyme, Nanjing, China). qRT-PCR was performed on a CFX96 real-time PCR system (Bio-Rad, Hercules, CA, United States) with AceQ qPCR SYBR Green Master Mix (Vazyme, Nanjing, China). PCR conditions were as follow: 95°C for 5 min, then 40 cycles of 95°C for 10 s, 60°C for 15 s, and 72°C for 30 s. Rice *18s rRNA* gene was used as an internal control to normalize data (Jain et al., 2006). Quantitative gene expression was analyzed by the 2^–ΔΔCT^ method and all primers used were listed in Supplementary Table S1.

### Yeast One-Hybrid (Y1H) Assays

Coding region of *ONAC066* was fused to GAL4 activation domain in effector vector pGADT7-Rec2 (Clontech, Mountain View, CA, United States). The promoter regions (1,500 bp upstream from ATG) of the genes of interest were amplified and cloned into reporter vector pHis2. pGADT7-Rec2-ONAC066 in combination with a reporter vector harboring the promoter of genes of interest were co-transformed into yeast strain Y187. DNA-protein interactions were verified by growth performance of yeasts grown on medium of SD/-Trp-Leu-His/3-amino-1,2,4-triazole (3-AT, a competitive inhibitor of His3 protein) (Clontech, Mountain View, CA, United States). All primers used for vector construction were listed in Supplementary Table S1.

### Gene Expression Profiling Analysis

Leave of 3-week-old *ONAC066*-OE11 and wild type (WT) plants grown in basal nutrient soil mixture under a 16 h light/8 h dark photoperiod at 28°C were collected and frozen in liquid nitrogen. Total RNA extraction, cDNA synthesis, purification, labeling, and chip hybridization were performed by CapitalBio Technology (Beijing, China) using the Affymetrix Rice GeneChip. Three independent biological replicates were used for each of the genotypes. After log_2_ transformation and normalization, genes whose expression showed fold change >2 (up-regulated) or <0.5 (down-regulated) with *q*-Value (a multiple-test corrected *p*-Value) (Benjamini and Hochberg, 1995) <5% were considered as differentially expressed genes. GO enrichment analysis of differentially expressed or co-expressed genes were performed using AgriGO website^1^ (Tian et al., 2017).

### Transient Expression Assays

Promoter regions (1500 bp from ATG codon) of *OsWRKY62*, *LOC_Os02g30110*, *LOC_Os06g37300* and *LOC_Os02g36150* were amplified and cloned into reporter vector pGreenII. pA7-GAL4BD and pA7-GAL4BD::ONAC066 were used as the effectors. Plasmids were extracted using CompactPrep Plasmid Midi Kit (Qiagen, Valencia, CA, United States). Protoplasts were prepared from rice mesophyll cells as previously described (Sun et al., 2012). Briefly, leaf pieces were infiltrated in a vacuum and gently rotated in a cell wall degrading enzyme mixture (0.4 M mannitol, 1.5% cellulose, and 0.3% macerozyme). After 4 h digestion, protoplasts were collected by filtering through 100 mm mesh. For co-transfection assays, 2 μg of the reporter plasmid and 2 μg of the effector plasmid were mixed and introduced into freshly prepared rice protoplasts under 20% PEG solution. After incubated for 12 h at 22°C in dark, LUC assays were performed using the Dual-Luciferase reporter assay system (Promega, Madison, WI, United States) according to the manufacturer’s instructions. The firefly luciferase (LUC) activity was normalized according to the humanized renilla LUC activity in each assay, and the relative ratio was determined by comparing with that of the empty vector. Averages of the relative ratios were calculated from three independent experiments and the primers used for LUC assay are listed in Supplementary Table S1.

### Chromatin Immuno-Precipitation (ChIP) and ChIP-PCR

Chromatin immuno-precipitation-PCR assays were performed as described previously (Chung et al., 2018). Briefly, chromatin was isolated from ∼3 g of leaves from 2-week-old *ONAC066*-OE11 plants. After fragment sonication, the DNA/protein complex was immune-precipitated with ChIP-grade antibody against GFP (Roche, Switzerland). After reverse cross-linking, the immunoprecipitated DNA was extracted with phenol/chloroform. The chromatin samples incubated with pre-immune (Pre) serum (GenScript, Nanjing, China) and before immunoprecipitation were used as negative controls and input controls, respectively. PCR was performed using specific primers (Supplementary Table S1) and the products were separated on 1.5% agarose gels and visualized by Goldview staining.

### Identification of Co-expressed Cytochrome P450 Genes

The co-expression data were downloaded from CREP (Collections of Rice Expression Profiling^2^) and PLANEX (Plant co-expression database^3^) databases comprising a total of 190 microarray experiments from diverse Chinese cultivated rice varieties and the publicly available GEO data from NCBI, respectively. The permutation test was done to determine the optimal threshold of the Pearson’s correlation coefficients (PCCs) (Butte et al., 2000; Carter et al., 2004). Absolute value of PCC>0.75 or 0.55 for CREP and PLANEX database, respectively, were considered as co-expressed genes with *ONAC066* (Aoki et al., 2007). Statistical significance for candidate genes in co-expression network construction was further determined by using a student *t*-test.

## Results

### *ONAC066* Contributes Positively to Rice Immunity Against *M. oryzae*

**FIGURE 1:**
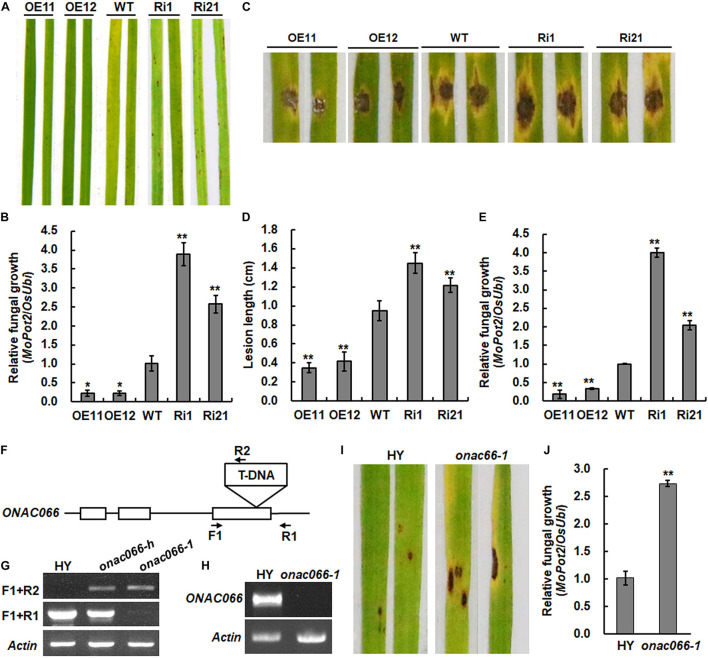
*ONAC066* positively regulates rice resistance against *Magnaporthe oryzae*. **(A,B)** Representative disease phenotype **(A)** and relative fungal growth **(B)** in leaves of *ONAC066*-OE and *ONAC066*-Ri plants. **(C–E)** Representative disease phenotype **(C)**, lesion length **(D)**, and relative fungal growth **(E)** in detached leaves of *ONAC066*-OE and *ONAC066*-Ri plants. **(F)** Gene structure of *ONAC066* and location of T-DNA. Open boxes indicate the exons while lines indicate introns. Primers used for genotyping are indicated. **(G)** Genotyping of *onac066-1* mutant by genome DNA PCR. F1 and R1 were used as gene-specific primers and R2/2707RB was a T-DNA-specific primer. *onac066-h* and *onac066-1* stand for heterozygous and homozygous rice plants, respectively. **(H)** Detection of *ONAC066* transcript in *onac066-1* mutant plants. **(I,J)** Representative disease phenotype **(I)** and relative fungal growth **(J)** in leaves of *onac066* plants. 4-week-old plants of different genotypes were inoculated by foliar spraying of spore suspension of *M. oryzae* **(A,I)** or detached leaves from 4-week-old plants were inoculated by dropping 5 μl spore suspension of *M. oryzae* **(C)**. Photographs and leaf samples were taken at 5 days post inoculation. Relative fungal growth was quantified by genomic qRT-RCR analyzing of the *M. oryzae MoPot2* and rice *OsUbi* genes and shown as ratios of *MoPot2*/*OsUbi*. Experiments were repeated at least three times with similar results, and results from one representative experiment are shown in panels **(A**,**C,I)**. Data presented in panels **(B,D,E,J)** are the means ± SD from three independent experiments and **/* indicated significant difference at *p* < 0.01 and *p* < 0.05 levels, respectively, by Student’s *t*-test, in comparison to WT.

To further explore the biological function of *ONAC066*, transgenic rice lines with overexpression of *ONAC066* or RNAi-mediated suppression of *ONAC066* were generated and two independent lines for overexpression (*ONAC066*-OE11 and *ONAC066*-OE12) and RNAi-mediated suppression (*ONAC066*-Ri1 and *ONAC066*-Ri21) were selected for this study (Yuan et al., 2019b). Involvement of *ONAC066* in rice immunity was examined by analyzing *M. oryzae*-caused disease phenotype on leaves of *ONAC066*-OE and *ONAC066*-Ri plants using whole plant inoculation and detached leaf inoculation assays. In whole plant inoculation assays, the *ONAC066*-OE11 and *ONAC066*-OE12 showed small, scattered lesions while *ONAC066*-Ri1 and *ONAC066*-Ri21 plants developed big and dark brown lesions, as compared to WT plants, which displayed typical blast lesions (Figure 1A). Fungal growth measurement results revealed a 22∼23% of reduction in *ONAC066*-OE plants and a 263∼396% of increase in *ONAC066*-Ri plants in comparison to that in WT plants (Figure 1B). In detached leaf inoculation assays, smaller lesions with a reduction of 37∼44% in lesion length were seen on detached leaves from *ONAC066*-OE plants while larger lesions with an increase of 28∼53% in lesion length were observed when compared with those in WT plants (Figures 1C,D). Similarly, the *ONAC066*-OE plants supported less fungal growth, accounting for 18∼33% of that in WT plants, while the *ONAC066*-Ri plants provided more fungal growth, representing 207∼403% of that in WT plants (Figure 1E). A mutant line *onac066-1*, in which T-DNA is inserted in the third exon of *ONAC066* gene (Figure 1F), was identified from RISD (Jeon et al., 2000; An et al., 2003) and homozygous plants were characterized by genotyping (Figure 1G). Transcript of *ONAC066* was undetectable in *onac066-1* plants (Figure 1H), suggesting that *onac066-1* is a null mutant. Disease assays indicated that the *onac066-1* plants showed more and larger disease lesions and supported a 1.5-fold higher level of fungal growth, as compared with WT plants (Figures 1I,J). Taken together, these results suggest that *ONAC066* acts as a positive regulator of rice immunity against *M. oryzae*.

### ONAC066 Has Transcriptional Activator Activity in Rice Cells

**FIGURE 2:**
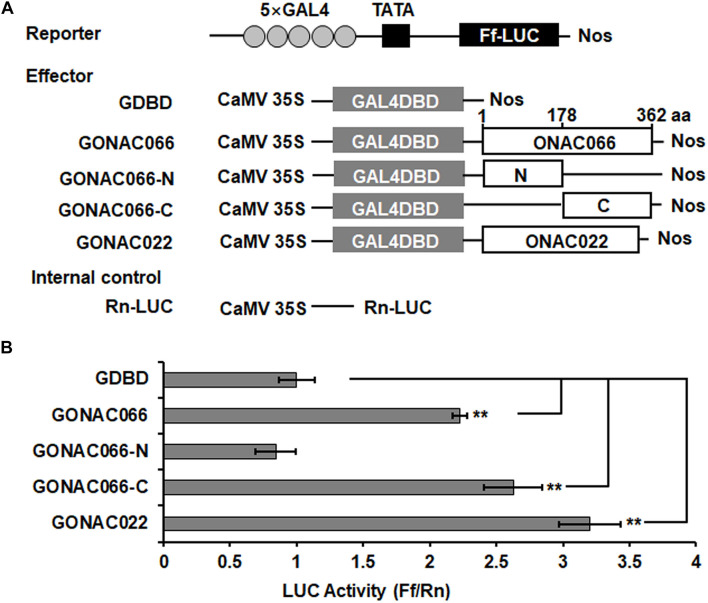
Transactivation activity of ONAC066 in rice protoplasts. **(A)** Diagrams of the effector and reporter constructs used in panel **(B)**. Amino acid positions in ONAC066 are indicated above the GONAC066 construct. **(B)** Luciferase (LUC) activity assay of GAL4DBD and GAL4 element-mediated LUC activity by full length or truncated ONAC066 in rice protoplasts. Data represented in panel **(B)** are the means ± SD from three independent experiments and ** indicated significant difference at *p* < 0.01 by two-tailed unpaired Student’s *t*-test.

We previously showed that ONAC066 has the binding ability to NACRS, AtJUB1 binding site (JBS) and JUB-like sequence and is a transcription activator in Y1H assays (Yuan et al., 2019b). The transcription activator activity of ONAC066 was further examined using rice protoplast transient expression system. The effector vectors used for this experiment contain either GAL4-BD alone (GDBD), or ONAC066 (GONAC066), ONAC066-N (GONAC066-N, containing the 1-178 aa N-terminal region), ONAC066-C (GONAC066-C, containing the 179-362 aa C-terminal region), or ONAC022 (GONAC022), closely related to ONAC066 (Hong et al., 2016; Yuan et al., 2019b), fused to GDBD (Figure 2A). In the rice protoplasts co-transfected with the effector and reporter vectors, the LUC activity of the effector GONAC066 or GONAC022 was increased by approximately 1.25- and 2.21-fold of the empty vector GDBD control (Figure 2B). Furthermore, the LUC activity of the effector GONAC066-N was similar to the empty vector GDBD control while the LUC activity of the effector GONAC066-C showed 1.64-fold higher than that of the empty vector GDBD control (Figure 2B). These results demonstrate that ONAC066 has transactivation activator activity in rice cells and also indicate that the C-terminus is critical for the transcriptional activator activity of ONAC066.

### ONAC066 Activates the Expression of Defense Genes

**FIGURE 3:**
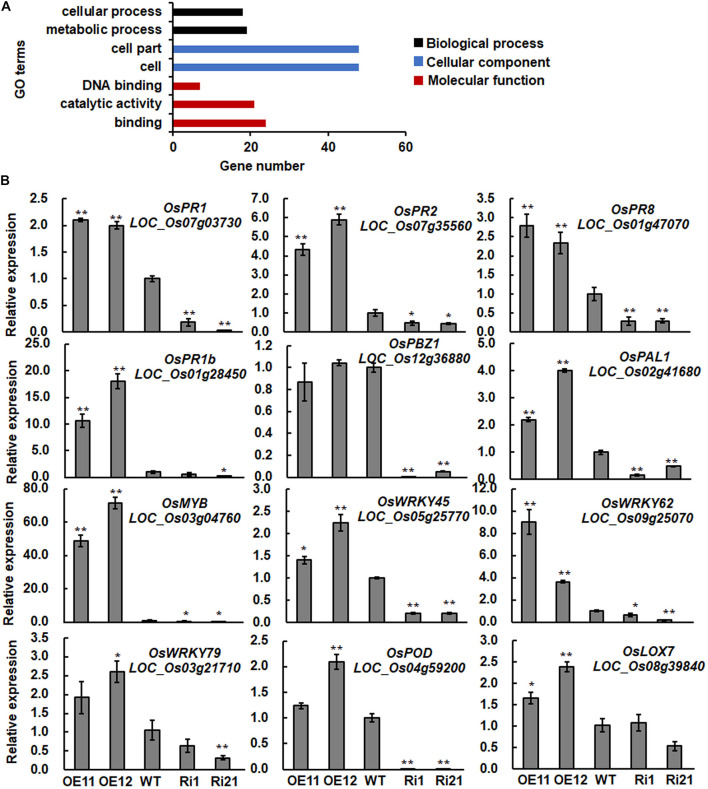
**(A,B)** Differentially expression of selected defense- and signaling-related genes in *ONAC066*-OE and *ONAC066*-Ri plants. Data represented are the means ± SD from three independent experiments and **/* indicated significant difference at *p* <0.01 and *p* < 0.05 levels, respectively, by Student’s *t*-test, in comparison to WT.

To explore the molecular mechanism of *ONAC066* in rice immunity and identify candidates for the ONAC066 target genes, gene expression profiling was performed and compared between *ONAC066*-OE11 and WT plants. After data processing, a total of 81 (*ONAC066*-OE11/WT fold changes >2 and *q*-Value < 5%) and 28 (*ONAC066*-OE11/WT fold changes <0.5 and *q*-Value < 5%) genes were found to be up- and down-regulated in *ONAC066*-OE11 plants grown under normal condition (Table 1 and Supplementary Table S2). The up-regulated and down-regulated genes in *ONAC066*-OE plants were clustered into 29 and four major categories, respectively (Figure 3A and Supplementary Table S3). The 7 main categories for up-regulated genes in *ONAC066*-OE plants belong to biological processes, cellular components, and molecular functions (Figure 3A). Notably, three genes for calmodulin-related proteins, LOC_Os03g59770, LOC_Os12g36910, and LOC_Os01g04280, were identified as ONAC066-up-regulated, and LOC_Os01g04280 shows 43.3% identity to Arabidopsis SARD1, which plays a critical role in salicylic acid (SA)-mediated defense signaling (Wang L. et al., 2011). LOC_Os02g50460 (OsPUB40) and LOC_Os03g13740 (OsPUB41) have 43.5 and 43.8% of identity, respectively, to tomato CMPG1, which is required for efficient activation of defense mechanisms in tomato (González-Lamothe et al., 2006). Among the up-regulated genes belonging to the DNA binding category, four WRKY TFs, including *OsWRKY45* (Shimono et al., 2007, 2012; Cheng et al., 2015), *OsWRKY62* (Peng et al., 2008; Fukushima et al., 2016; Liu et al., 2016), and *OsWRKY76* (Yokotani et al., 2013; Liu et al., 2016), which have been shown to be involved in rice immunity, were identified (Table 1). In addition, three defense genes encoding for PR1, PR2 (β-1,3-glucanase), and PR8 (chitinase) were also up-regulated in *ONAC066*-OE plants (Table 1).

**TABLE 1:** Up-regulated genes in ONAC066-OE11 plants.

TIGR ID	Description	Fold changes^a^ (OE11/WT)	*q*-Value (%)^b^ (OE11/WT)	FR13^c^ 3 dpi	FR13^c^ 4 dpi
**Transcription factors**					
LOC_Os03g04760	Myb protein	25.25	0	1.00	0.99
LOC_Os09g25060	OsWRKY76	3.40	0	2.15	3.31
LOC_Os03g21710	OsWRKY79	2.39	1.10	1.13	1.26
LOC_Os09g25070	OsWRKY62	2.39	0.33	1.19	3.02
LOC_Os05g25770	OsWRKY45	2.03	0	1.40	1.20
**Protein kinases**					
LOC_Os11g10710	Protein kinase	8.13	0	1.05	1.66
LOC_Os10g04520	Protein kinase	3.25	0	1.18	2.08
LOC_Os09g29540	OsWAK82	2.39	0	2.04	0.73
Os11g0470200	Receptor-like kinase	2.26	4.14	0.90	0.87
LOC_Os09g29520	Protein kinase	2.15	0	1.40	1.38
LOC_Os10g04450	Protein kinase	2.10	0	0.80	0.75
LOC_Os02g13780	LRR receptor-like kinase	2.08	2.93	1.00	2.32
*Domain unknown functions (DUF)*					
Os07g0162450	DUF3778	7.77	0	1	1
LOC_Os12g36750	DUF231	5.43	0	0.99	1.28
LOC_Os07g03040	DUF1719	3.55	0	1.71	1.28
LOC_Os07g27350	DUF1446	3.25	0	0.86	1.18
LOC_Os04g42610	DUF869	2.31	0.33	0.96	0.85
LOC_Os03g08880	DUF250/purine permease 3	2.25	3.83	1.00	0.99
LOC_Os12g33300	DUF6	2.03	0.33	1.88	3.22
**Oxidation**					
LOC_Os11g42220	Laccase-20	4.74	3.18	0.81	0.88
LOC_Os04g41810	Ferric reductase	2.00	2.54	1.00	0.99
LOC_Os04g59200	Peroxidase	2.09	0.33	1.05	3.21
LOC_Os08g39840	Lipoxygenase 7	2.12	0	1.75	2.27
**Enzymes**					
LOC_Os08g14190	Sulfotransferase 3	2.81	0	1.11	0.80
LOC_Os10g39260	Aspartic proteinase	2.64	0.50	1.14	2.05
LOC_Os05g08480	UDP-glycosyltransferase	2.59	0	1.04	1.22
LOC_Os04g47360	Endopeptidase	2.45	3.18	1.54	1.33
LOC_Os07g36560	Transferase	2.08	3.18	1.80	3.50
LOC_Os12g43970	Hydrolase	2.06	0	1.48	2.08
LOC_Os08g16260	Cytochrome P450	9.37	3.18	1.00	1.00
LOC_Os06g45960	Cytochrome P450	3.19	0	1.32	2.09
**PR genes**					
LOC_Os01g47070	PR8/chitinase III/OsChib3a	7.51	0	1.50	1.95
LOC_Os07g03730	Pathogenesis-related protein 1	2.19	2.91	1.54	2.44
LOC_Os07g35560	PR2/β-1,3-glucanase	2.04	1.06	2.56	2.60
LOC_Os07g043220	Thionin-like peptide	2.37	0	0.93	0.73
**Calmodulin related**					
LOC_Os03g59770	Calmodulin-like protein 2	2.33	3.36	3.04	2.45
LOC_Os12g36910	CBP60g	2.23	0	2.23	2.53
LOC_Os01g04280	SARD1	2.22	0	2.30	3.12
**Transporters**					
LOC_Os01g70490	K^+^ potassium transporter 5	3.83	0	1.37	2.29
LOC_Os03g62200	NH_4__+_ transporter 3 member 2	2.13	0	1.41	2.61
**U-box proteins**					
LOC_Os02g50460	U box protein OsPUB40	3.17	0	1.48	2.32
LOC_Os03g13740	U box protein OsPUB41	2.09	0	1.61	3.77
**Others**					
LOC_Os09g30320	BURP15	293.25	0	1.00	1.00
LOC_Os01g36850	Transposon protein	6.59	0	1.00	0.99
LOC_Os10g41838	HAT domain protein	5.55	1.06	1.00	0.92
LOC_Os11g17954	Transposon protein	5.45	0	1.00	0.99
LOC_Os03g50670	Retrotransposon protein	4.44	0	1.00	0.97
LOC_Os06g45970	OsSAUR26	4.37	0	2.43	3.58
LOC_Os10g37840	COMPLEX C SUBUNIT B	2.44	0	1.00	0.87
LOC_Os01g50100	Multidrug resistance protein 1	2.32	4.89	1.86	2.58
Os12g0113600	Hypothetical protein	9.55	0	0.90	0.88
Os06g0318533	Hypothetical protein	6.47	0	0.91	1.05
Os01g0606400	Hypothetical protein	5.29	0	0.81	1.04
	Hypothetical protein	4.77	0	2.73	1.39
LOC_Os05g30700	Hypothetical protein	4.65	0	0.88	1.23
LOC_Os07g01904	Hypothetical protein	4.56	0	0.86	1.12
Os06g0685400	Hypothetical protein	4.29	1.06	1.00	0.98
Os02g0545700	Hypothetical protein	3.76	0	1.00	1.05
Os07g0429700	Hypothetical protein	3.54	1.80	1.00	0.98
LOC_Os03g03724	Hypothetical protein	3.48	0.93	0.91	1.078
Os04g0280250	No hits	3.26	0	1.05	1.20
LOC_Os07g01890	Hypothetical protein	3.08	0	0.93	1.02
Os06g0579100	Hypothetical protein	3.04	0	2.03	1.25
Os10g0352200	Hypothetical protein	3.01	0	1.00	0.99
Os11g0624600	No hits	2.94	0	1.08	1.06
LOC_Os08g15710	Hypothetical protein	2.58	0	1.47	0.96
Os08g0240533	No hits	2.49	2.39	1.00	1.16
LOC_Os08g20220	Hypothetical protein	2.46	0	0.74	0.96
Os01g0852800	No hits	2.38	0	1.00	1.06
LOC_Os05g01010	Hypothetical protein	2.37	1.06	1.01	0.99
Os10g0134400	No hits	2.35	2.08	2.07	1.78
LOC_Os01g49750	Hypothetical protein	2.33	0.93	0.97	1.15
Os11g0422000	Hypothetical protein	2.33	2.89	0.94	1.48
LOC_Os02g47390	Hypothetical protein	2.23	0	1.00	1.00
Os06g0584400	Hypothetical protein	2.23	0	1.00	1.01
Os11g0471200	Hypothetical protein	2.20	2.26	1.00	1.087
LOC_Os10g07160	Hypothetical protein	2.20	3.36	1.06	1.27
Os02g0501350	Hypothetical protein	2.17	0.50	1.11	1.12
LOC_Os01g09190	Hypothetical protein	2.08	3.18	1.00	1.03
Os12g0191601	Hypothetical protein	2.05	0	2.02	1.51
LOC_Os06g14870	Hypothetical protein	2.02	1.06	0.97	0.99

*^*a*^Averages of fold changes from three independent biological samples were shown.*

*^*b*^*q*-Value (%) shows the significance analysis based on three independent biological replicates. The genes with a cutoff *q*-Value < 5% and fold changes >2 were defined as differentially expressed genes.*

*^*c*^Fold changes (*M. oryzae* FR13-infected/mock control) were retrieved from microarray dataset GSE7256 (Ribot et al., 2008), and genes with >2-fold changes were considered as up-regulated genes (shaded data).*

Publicly available microarray data GSE7256 (Ribot et al., 2008), which was generated from 2-week-old cv. Nipponbare plants infected with *M. grisea* virulent isolate FR13, was used to examine whether the up-regulated genes in *ONAC066*-OE11 plants are also responsive to *M. oryzae*. Among the 81 up-regulated genes in *ONAC066*-OE plants, 26 genes (32% of the up-regulated genes), were up-regulated in rice plants at 3 and/or 4 dpi after infection by *M. oryzae* isolate FR13 (Table 1). It is thus likely that overexpression of *ONAC066* activates the expression of a set of genes that are responsive to *M. oryzae*.

To verify the expression patterns of the up-regulated genes in *ONAC066*-OE plants, we further compared the expression levels of 12 genes (9 ONAC066-up-regulated genes and 3 previously identified defense genes) among WT, *ONAC066*-OE and *ONAC066*-Ri plants by qRT-PCR. The expression levels of the ONAC066-up-regulated genes *OsPR1*, *OsPR2*, *OsPR8*, *OsWRKY45*, *OsWRKY62*, *OsWRKY79*, *Myb* (LOC_Os03g04760), *POD* (LOC_Os04g59200), and *OsLOX7* (LOC_Os08g39840) as well as of two well-known defense genes *OsPR1b* and *OsPAL1* (Inui et al., 1997; Agrawal et al., 2001) were significantly up-regulated in *ONAC066*-OE plants but markedly down-regulated in *ONAC066*-Ri plants (Figure 3B). Expression of another well-known defense gene *OsPBZ1* (Midoh and Iwata, 1996) was significantly down-regulated in *ONAC066*-Ri plants but not changed in *ONAC066*-OE plants (Figure 3B). In the transcriptomic analyses, no expression data for *OsPR1b* were obtained and *OsPBZ1* and *OsPAL1* were not considered as differentially expressed genes as their expression was up-regulated by ∼1 fold with *q*-Value > 5%. The difference in expression change of *OsPAL1* in ONAC066-OE plants, as revealed by qRT-PCR and transcriptomic analyses, may be due to different techniques used. Collectively, these data indicate that *ONAC066* plays a role in activation of defense signaling and response in rice immunity.

### ONAC066 Directly Activates Expression of *OsWRKY62*

**FIGURE 4:**
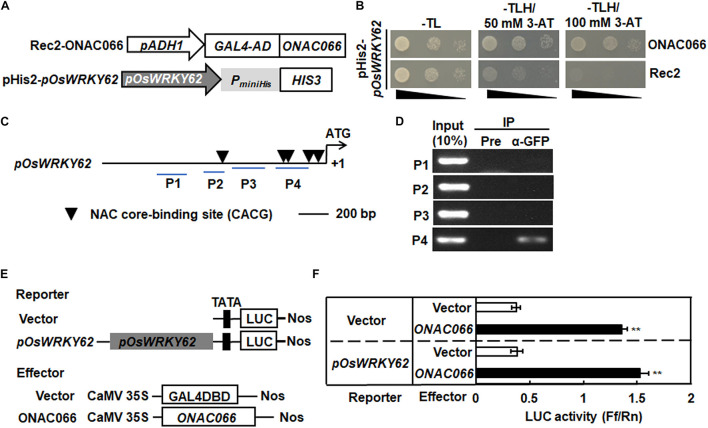
ONAC066 activates *OsWRKY62* promoter. **(A)** Diagram of yeast expression construct Rec2-*ONAC066* and reporter construct pHis2-*pWRKY62*. **(B)** Growth performance of transformants on SD/-Leu-/Trp/-His medium containing 50 or 100 mM 3-AT. **(C)** Diagram of putative NAC core-binding sites in *OsWRKY62* promoter. P1-P4 were the probes used in ChIP-PCR assays. **(D)** ChIP-PCR analysis of ONAC066 binding to *OsWRKY62* promoter. ChIP of ONAC066-GFP transgenic line using GFP antibody (α-GFP) or pre-immune (Pre) serum was performed and precipitated DNA fragments were subject to PCR analysis using *OsWRKY62* promoter primers. 10% of chromatin amount before IP was used as positive controls (input) and IP sample with pre-immune serum was used as a negative control. **(E,F)** Activation of *OsWRKY62* promoter by ONAC066. **(E)** Diagrams of the effector and reporter constructs. A 1500 bp from start codon was used as the *OsWRKY62* promoter. **(F)** Luciferase activity in protoplasts co-transfected with the reporter and different combinations of effectors was determined. Experiments were repeated at least three times with similar results, and results from one representative experiment are shown in panels **(B,D)**. Data presented in panel **(F)** are the means ± SD from three independent experiments and ** indicated significant difference at *p* < 0.01 by Student’s *t*-test, in comparison to empty vector.

The up-regulation pattern of *OsWRKY45*, *OsWRKY62*, *OsWRKY76*, and *OsWRKY79* in *ONAC066*-OE plants led to examine whether these WRKY genes were ONAC066 targets. The binding ability of ONAC066 to the promoters of these WRKY genes was examined by Y1H assays. For this purpose, the 1500 bp promoter regions from start codons of these WRKY genes were amplified and cloned into pHis2 vectors (Figure 4A) and were then co-transformed with vector Rec2-ONAC066 into yeast strain Y187. Yeast co-transformed with Rec2-ONAC066 and pHis2-*pWRKY62* grew normally on 3-AT containing medium, while yeast co-transformed with empty Rec2 and pHis2 vectors did not grow well at a high concentration of 3-AT (Figure 4B), suggesting that ONAC066 could directly bind to the promoter of *OsWRKY62*. However, growth performance of yeasts co-transformed with Rec2-ONAC066 and pHis2-*pWRKY45*, pHis2-p*WRKY76*, or pHis2-*pWRKY79* was indistinguishable to yeast co-transformed with empty Rec2 and pHis2 vectors on 3-AT containing medium (Supplementary Figure S1), indicating that ONAC066 did not bind to the promoters of *OsWRKY45*, *OsWRKY76*, and *OsWRKY79*.

The binding site of ONAC066 in *OsWRKY62* promoter was further mapped by ChIP-PCR. Four NAC core-binding sites (CACG) (Tran et al., 2004) were identified within the 1500 bp promoter region of the *OsWRKY62* gene and four probe regions were chosen for ChIP-PCR assays (Figure 4C). No PCR amplicon was detected in the four probe regions when pre-immune serum was used in IP (Figure 4D). A clear amplicon was observed in P4 probe region and no amplicon was detected in P1, P2, and P3 probe regions, when anti-GFP antibody was used in IP (Figure 4D), demonstrating that ONAC066 binds to P4 region, which contains three NAC core-binding sites (Figure 4C), in the *OsWRKY62* promoter.

The ability of ONAC066 to drive the transcription of *OsWRKY62* was further explored by using the rice protoplast transient expression system with the reporter vector harboring *OsWRKY62* promoter, and the effector vector expressing *ONAC066* (Figure 4E). In rice protoplasts co-transfected with the effector vector *ONAC066* and reporter vector *pOsWRKY62*, the LUC activity was significantly higher than that in rice protoplasts co-transfected with the empty effector vector and reporter vector pOsWRKY62 (Figure 4F). These results indicate that ONAC066 transcriptionally drives the *OsWRKY62* promoter. Taken together, it is clear that ONAC066 can bind to the NAC core-binding sites in *OsWRKY62* promoter and thus drives the expression of *OsWRKY62*.

### ONAC066 Modulates Expression of Three P450 Genes

**FIGURE 5:**
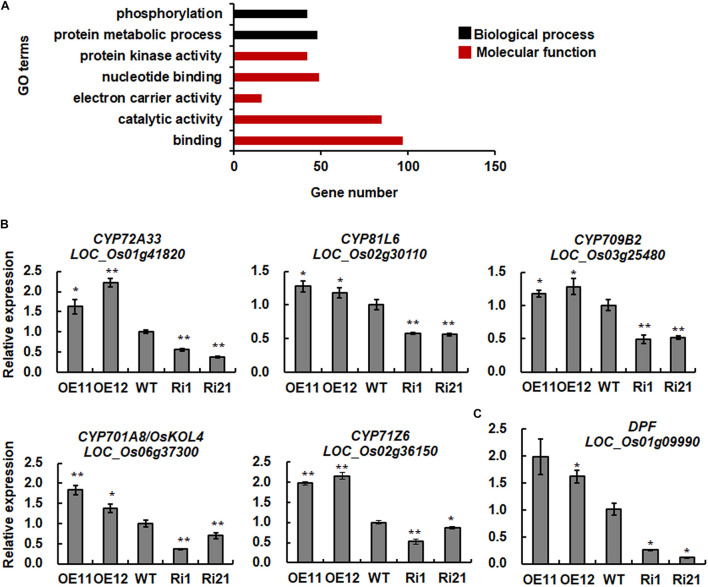
Enriched GO terms of genes co-expressed with ONAC066 and expression of the selected cytochrome P450 genes and *DPF* in *ONAC066*-OE and *ONAC066*-Ri plants. **(A)** Enriched GO terms of genes co-expressed with ONAC066. **(B)** Expression of selected cytochrome P450 genes. **(C)** Expression of *DPF*. Data represented in panels **(B,C)** are the means ± SD from three independent experiments and **/* indicated significant difference at *p* < 0.01 and *p* < 0.05 levels, respectively, by Student’s *t*-test, in comparison to WT.

To further characterize putative ONAC066 targets, *ONAC066* was used as a guide gene to identify its co-expressed genes using expression profiling data from 190 datasets in CREP and NCBI GEO-based PLANEX databases. As results, 86 and 114 genes with PCC values greater than 0.75 or 0.55 for CREP and PLANES databases, respectively, were identified as tightly co-expressed genes with *ONAC066* (Supplementary Tables S4, S5). Enriched GO annotations were particularly clustered into categories of biological process and molecular functions, which are tightly associated with catalytic activity, binding, electron carrier activity, transcription regulator and transporter activity (Figure 5A).

A total of 15 co-expressed genes were simultaneously identified in CREP and PLANEX databases (Table 2), among which 5 belong to the cytochrome P450 gene family. In fact, 14 cytochrome P450 genes were found to be co-expressed with *ONAC066* in CREP and PLANEX databases and five of them were also induced by *M. oryzae* FR13 (Table 3). To verify the co-expression of these cytochrome P450 genes with *ONAC066*, we compared their expression patterns of 5 cytochrome P450 genes among WT, *ONAC066*-OE and *ONAC066*-Ri plants by qRT-PCR. The expression levels of these 5 cytochrome P450 genes, including *LOC_Os01g41820* (CYP72A33), *LOC_Os02g30110* (CYP81L6), *LOC_Os03g25480* (CYP709B2), *LOC_Os06g37300* (CYP701A8/OsKOL4), and *LOC_Os02g36150* (CYP71Z6), were significantly up-regulated in *ONAC066*-OE plants but markedly down-regulated in *ONAC066*-Ri plants (Figure 5B), further confirming the co-expression feature of these cytochrome P450 genes with *ONAC066*. However, the expression patterns of the remaining co-expressed cytochrome P450 genes in *ONAC066*-OE and *ONAC066*-Ri plants were similar to WT (Supplementary Figure S2). Moreover, *DPF*, encoding a basic helix-loop-helix transcription factor acting as a master transcription factor in biosynthesis of diterpenoid phytoalexins (Yamamura et al., 2015), was also significantly up-regulated in *ONAC066*-OE plants but markedly down-regulated in *ONAC066*-Ri plants (Figure 5C).

**TABLE 2:** Co-expressed genes simultaneously identified in CREP and PLANEX databases.

TIGR locus ID	Description	PCC^a^	PCC^a^	FR13 3 dpi^b^	FR13 4 dpi^b^
LOC_Os01g18584	OsWRKY9	0.91	0.56	1.17	1.25
LOC_Os02g19550	Lectin receptor-type protein kinase	0.90	0.59	1.14	1.42
LOC_Os01g70210	Phosphatidylinositol transfer protein	0.89	0.56	1.00	1.45
LOC_Os07g38800	Lectin-like receptor kinase	0.88	0.64	1.70	1.40
LOC_Os07g44130	Cytochrome P450 72A1	0.87	0.60	1.17	1.08
LOC_Os06g07200	Syntaxin 132	0.86	0.63	1.36	1.70
LOC_Os07g38810	Lectin receptor-type protein kinase	0.85	0.68	1.23	1.42
LOC_Os01g41820	Cytochrome P450 72A1	0.84	0.57	0.99	1.22
LOC_Os06g18820	Expressed protein	0.83	0.58	1.08	0.93
LOC_Os04g15580	Serine threonine-protein kinase	0.80	0.57	1.45	1.73
LOC_Os01g59000	Cytochrome P450 94A2	0.79	0.57	1.17	1.59
LOC_Os02g30110	Cytochrome P450	0.78	0.56	1.06	1.24
LOC_Os01g14590	Pathogen-related protein	0.76	0.57	0.99	0.93
LOC_Os01g52790	Cytochrome P450 72A1	0.76	0.62	1.46	2.78
LOC_Os12g25170	Disease resistance protein RGA2	0.76	0.65	1.28	1.89

*^*a*^Pearson’s correlation coefficients value of the co-expressed genes identified from CREP and PLANEX databases, respectively.*

*^*b*^Fold changes (*M. oryzae* FR13-infected/mock control) were retrieved from microarray dataset GSE7256 (Ribot et al., 2008), and genes with >1.5-fold changes were considered as up-regulated genes (shaded data).*

**TABLE 3:** Cytochrome P450 genes co-expressed with ONAC066.

TIGR ID	Annotations	PCC^a^	FR13^b^ 3 dpi	FR13^b^ 4 dpi
**CREP database** ^c^				
**LOC_Os07g44130** ^d^	**Cytochrome P450 72A1**	0.86	1.36	1.70
**LOC_Os01g41820**	**Cytochrome P450 72A1**	0.83	1.72	1.69
LOC_Os10g36848	Cytochrome P450 84A1	0.79	0.98	1.32
LOC_Os03g39690	Cytochrome P450 71D7	0.79	0.98	2.46
**LOC_Os01g59000**	**Cytochrome P450 94A2**	0.79	1.81	1.68
LOC_Os10g30410	Cytochrome P450 71D7	0.78		
**LOC_Os02g30110**	**Cytochrome P450 81L6**	0.78	0.96	0.76
LOC_Os01g43851	Cytochrome P450 72A1	0.77		
**LOC_Os01g52790**	**Cytochrome P450 72A1**	0.76	1.28	1.89
LOC_Os02g32770	Cytochrome P450 71D6	0.76		
LOC_Os03g25480	Cytochrome P450 72A1	0.75	1.06	0.82
**PLANEX database** ^e^				
LOC_Os06g37300	Cytochrome P450 701A8/OsKOL4	0.64	1.15	1.48
LOC_Os02g36190	Cytochrome P450 71Z7	0.62	1.94	2.22
**LOC_Os01g52790**	**Cytochrome P450 72A14**	0.61	1.28	1.89
**LOC_Os07g44130**	**Cytochrome P450 709B2**	0.60	1.36	1.70
**LOC_Os01g41820**	**Cytochrome P450 72A15**	0.57	1.72	1.69
**LOC_Os01g59000**	**Cytochrome P450 94D2**	0.57	1.81	1.68
**LOC_Os02g30110**	**Cytochrome P450 81D5**	0.56	0.96	0.76
LOC_Os02g36150	Cytochrome P450 71Z6	0.56	1.03	1.03

*^*a*^Pearson’s correlation coefficients.*

*^*b*^Fold changes (*M. oryzae*-infected/mock control) were retrieved from microarray dataset GSE7256 (Ribot et al., 2008), and genes with >1.5-fold changes were considered as up-regulated genes (shaded data).*

*^*c*^Data from http://crep.ncpgr.cn/crep-cgi/home.pl.*

*^*d*^CYP genes in bold were found in two databases.*

*^*e*^Data from http://planex.plantbioinformatics.org.*

**FIGURE 6:**
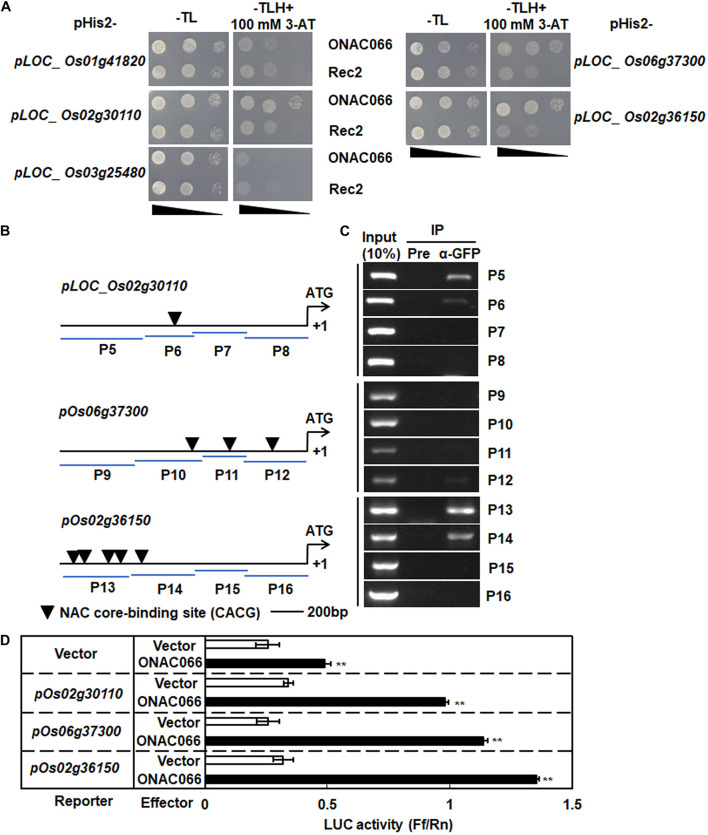
ONAC066 activates promoters of three cytochrome P450 genes. **(A)** Growth performance of transformants on SD/-Leu-/Trp/-His medium containing 100 mM 3-AT. **(B)** Diagrams of putative NAC core-binding sites in the promoters of the selected cytochrome P450 genes. P5-P16 represent the probes used in ChIP-PCR assays. **(C)** ChIP-PCR analysis of ONAC066 binding to promoters of the selected cytochrome P450 genes. ChIP of ONAC066-GFP transgenic line using GFP antibody (α-GFP) or pre-immune (Pre) serum was performed and precipitated DNA fragments were subject to PCR analysis using different primers for promoters of the selected cytochrome P450 genes. 10% of chromatin amount before IP was used as positive controls (input) and IP sample with pre-immune serum was used as a negative control. **(D)** Activation of promoters of the selected cytochrome P450 genes by ONAC066. Fragments of 1500 bp from start codon were used as promoters of the selected cytochrome P450 genes. Luciferase activity in protoplasts co-transfected with the reporter and different combinations of effectors was determined. Experiments were repeated at least three times with similar results, and results from one representative experiment are shown in panels **(A,C)**. Data presented in panel **(D)** are the means ± SD from three independent experiments and ** indicated significant difference at *p* < 0.01 by Student’s *t*-test, in comparison to empty vector.

The co-expression feature of the cytochrome P450 genes with *ONAC066* raised the possibility that they were ONAC066 targets. The binding ability of ONAC066 to promoters of the co-expressed cytochrome P450 genes was therefore examined by Y1H assays. For this purpose, the 1500 bp promoter regions from start codons of these cytochrome P450 genes were cloned into pHis2 vectors and were then co-transformed with vector Rec2-*ONAC066* into yeast strain Y187. Yeast co-transformed with Rec2-ONAC066 and pHis2-*pLOC_Os02g30110* or pHis2-*pLOC_Os06g37300* or pHis2-*pLOC_Os02g36150* grew well on SD/-Trp/-Leu/-His medium containing 100 mM 3-AT, while yeasts co-transformed with Rec2-*ONAC066* and pHis2-*pLOC_Os01g41820* or pHis2-*pLOC_Os03g25480* did not grow on the same medium (Figure 6A). These results indicate that ONAC066 could bind to the promoters of *LOC_Os02g30110*, *LOC_Os06g37300*, and *LOC_Os02g36150* to activate the reporter gene expression in yeasts.

The binding site of ONAC0066 in the promoters of *LOC_Os02g30110*, *LOC_Os06g37300*, and *LOC_Os02g36150* was further mapped by ChIP-PCR. One, three and five NAC core-binding sites (CACG) (Tran et al., 2004) were identified within the 1500 bp promoter regions of *LOC_Os02g30110*, *LOC_Os06g37300* and *LOC_Os02g36150*, respectively, and four probe regions for each promoter of the genes were chosen for ChIP-PCR assays (Figure 6B). No PCR amplicon was detected in the four probe regions for each promoter of the genes tested when pre-immune serum was used in IP (Figure 6C). Amplicons were observed in P5 and P6 probe regions in *LOC_Os02g30110* promoter, P12 probe region in *LOC_Os06g37300* promoter and P13 and P14 probe regions in *LOC_Os02g36150* promoter, when anti-GFP antibody was used in IP (Figure 6C). Notably, a clear amplicon was detected in P5 probe region of *LOC_Os02g30110* promoter, which does not contain a NAC core-binding site (Figure 6C). Together, these results suggest that ONAC066 binds to the *LOC_Os02g30110*, *LOC_Os06g37300*, and *LOC_Os02g36150* promoters.

The ability of ONAC066 to drive the transcription of *LOC_Os02g30110*, *LOC_Os06g37300*, and *LOC_Os02g36150* genes was further explored by using the rice protoplast transient expression system with the effector vector expressing *ONAC066* and the reporter vector harboring the promoters of *LOC_Os02g30110*, *LOC_Os06g37300*, or *LOC_Os02g36150*. In rice protoplasts co-transfected with the effector vector *ONAC066* and reporter vector *pOs02g30110*, *pOs06g37300*, or *pOs02g36150*, the LUC activity was significantly higher than that in rice protoplasts co-transfected with the empty effector vector and reporter vectors (Figure 6D). These results indicate that ONAC066 transcriptionally drives the promoters of the *LOC_Os02g30110*, *LOC_Os06g37300* and *LOC_Os02g36150* genes. Collectively, it is clear that ONAC066 can bind to the promoters of *LOC_Os02g30110*, *LOC_Os06g37300* and *LOC_Os02g36150* and thus drives their expression.

## Discussion

The present study further investigated the function and explored the molecular mechanism of *ONAC066* in rice immunity. ONAC066 belongs to ONAC022 subgroup (Ooka et al., 2003) and its Arabidopsis homologous ANAC042 was found to suppress defense responses to a bacterial pathogen (Shahnejat-Bushehri et al., 2016). *ONAC066* transcriptionally responded to *M. oryzae* infection and abiotic stress factors (Liu et al., 2018; Yuan et al., 2019b) and thus is a stress-responsive NAC TF that can respond to multiple abiotic and biotic factors in rice. A recent study revealed that *ONAC066* positively regulates resistance to blast and bacterial blight diseases through ABA-dependent signaling pathway (Liu et al., 2018). In this study, *ONAC066*-OE plants exhibited enhanced resistance while *ONAC066*-Ri and *onac066-1* plants showed attenuated resistance to *M. oryzae* (Figure 1), clearly demonstrating that ONAC066 plays a positive role in rice immunity against *M. oryzae*. Furthermore, we previously demonstrated that ONAC066 functions as a positive regulator of drought and oxidative stress response in rice (Yuan et al., 2019b). Collectively, these findings enable *ONAC066* as a promising factor to be used in creating novel rice germplasm with improved abiotic stress tolerance and disease resistance.

ONAC066 is a nucleus localized TF with an ability to bind to a canonical NAC recognition sequence (Tran et al., 2004) and a newly identified *cis*-element JUB1 binding site (Wu et al., 2012) in yeasts (Liu et al., 2018; Yuan et al., 2019b). ONAC066 has transcription activator activity that depends on its C-terminal in yeast (Yuan et al., 2019b). This is further demonstrated in the present study that ONAC066 has transcription activator activity in rice cells (Figure 2). Therefore, ONAC066 acts as a transcription activator and exerts its biological function through activating downstream target genes. Notably, the C-terminal region of ONAC066 was capable of initiating the transcription in rice cells (Figure 2), which is similar to the previous observations on ONAC022 and OsNAP, whose C-terminal regions showed abilities to activate the transcription in yeast (Liang et al., 2014; Hong et al., 2016). Generally, the N-terminals of NAC proteins recognize their *cis*-element sequences while the C-terminals is responsible to initiate the transcription of the downstream target genes (Olsen et al., 2005). Whether the C-terminal of ONAC066 affect or abolish its interactions with the *cis*-elements in *OsWRKY62* promoter is unknown. However, our previous study showed that the full ONAC022 with its C-terminal could bind to the NACRS element in electrophoretic mobility shift assays (Hong et al., 2016), implying that the C-terminals may not affect the interactions of NAC proteins with the *cis*-elements in promoters of their target genes. On the other hand, ONAC066 was found to bind to probe regions harboring NAC core-binding sites in the promoters of *OsWRKY62*, *LOC_Os02g30110*, *LOC_Os06g37300*, and *LOC_Os02g36150* genes (Figures 4, 6). However, ONAC066 also bound to a probe region without a NAC core-binding site in *LOC_Os02g30110* promoter (Figure 6), suggesting that ONAC066 can bind to other unknown *cis*-elements in the promoters of its target genes. This is partially supported by our previous observation that ONAC066 not only bound to NACRS and JBS *cis*-elements in Y1H assays but also bound to a JBS-like *cis*-element in *OsDREB2A* promoter in ChIP-PCR and EMSA assays (Yuan et al., 2019b). Although the interaction of ONAC066 with *OsWRKY62* promoter was validated by different experiments in the present study, including Y1H, ChIP-PCR and rice protoplast transient expression assays, the detailed *cis*-element(s) in *OsWRKY62* promoter for ONAC066 need to be further identified.

Because ONAC066 has transcription activator activity in rice cells (Figure 2), we thus sought for genes that were up-regulated in *ONAC066*-OE plants by gene expression profiling analyses between *ONAC066*-OE11 and WT plants. Surprisingly, only 81 up-regulated genes were identified in *ONAC066*-OE plants (Table 1). The number of the up-regulated genes in *ONAC066*-OE plants is comparable to the numbers of co-expressed genes identified in two different databases (Supplementary Tables S4, S5). The up-regulated expression of genes for PRs, WRKYs and homologues of immunity-related factors (SARD1 and CMPG1) demonstrates the activation of defense response in *ONAC066*-OE plants. Importantly, 26 of these up-regulated genes were predicted to be induced by *M. oryzae* infection (Table 1), suggesting that ONAC066 may modulate a group of genes that are responsive to *M. oryzae*. Defense and signaling genes were significantly up-regulated in *ONAC066*-OE plants but down-regulated in *ONAC066*-Ri plants (Figure 3), indicating that the expression of these defense and signaling genes depends largely on ONAC066 function. This is partially similar to OsNAC111, which positively contributes to disease resistance by regulating the expression of a specific set of defense genes in disease response (Yokotani et al., 2014). Unlike OsNAC111 whose suppression did not affect rice immunity, *ONAC066*-Ri plants showed increased susceptibility to *M. oryzae* infection (Figure 1), implying that ONAC066 is required for basal immunity against *M. oryzae*.

Although the immunity-related functions of rice NAC TFs have been well documented (Nakashima et al., 2007; Kaneda et al., 2009; Yoshii et al., 2009, 2010; Sun et al., 2013; Yokotani et al., 2014; Park et al., 2017; Liu et al., 2018; Wang et al., 2018), the molecular mechanisms of these NAC TFs to regulate rice immunity are largely unknown yet. ONAC066 was previously found to directly bind to the promoter of *OsNCED4* to modulate its expression (Liu et al., 2018). OsNCED4 is one of the 9-*cis*-epoxycarotenoid dioxygenases acting as a rate-limiting enzyme for abscisic acid biosynthesis (Huang et al., 2018, 2019; Hwang et al., 2018). Considering that ONAC066 is a transcription activator (Figure 2), the binding of ONAC066 to *OsNCED4* promoter should drive the expression of *OsNCED4*, which in turn leads to ABA biosynthesis. However, the expression level of *OsNCED4* was significantly down-regulated and ABA level was also markedly reduced in *ONAC066*-overexpressing plants (Liu et al., 2018). Therefore, whether *OsNCED4* is a true target of ONAC066 and whether ABA signaling is involved in ONAC066-mediated regulation of rice immunity need to be further examined. On the other hand, SARD1 has been demonstrated to play a critical role in SA signaling through regulating pathogen-induced biosynthesis of SA in Arabidopsis (Zhang et al., 2010; Wang L. et al., 2011). The up-regulated expression of *LOC_Os01g04280*, which shows 43.3% of identity to Arabidopsis SARD1, in *ONAC066*-OE plants raises a possibility that ONAC066 may regulate rice immunity through SA signaling via modulating *LOC_Os01g04280* expression.

In the present study, immunity-related WRKY genes including *OsWRKY45* and *OsWRKY62* (Shimono et al., 2007, 2012; Peng et al., 2008; Yokotani et al., 2013; Cheng et al., 2015; Fukushima et al., 2016; Liu et al., 2016) were significantly up-regulated in *ONAC066*-OE plants but down-regulated in *ONAC066*-Ri plants (Figure 3 and Table 1). Y1H assays revealed ONAC066 could bind the *OsWRKY62* promoter but not the promoters of *OsWRKY45*, *OsWRKY76* and *OsWRKY79* (Figure 4A and Supplementary Figure S1). ChIP-PCR and rice protoplast transient expression assays demonstrated that ONAC066 bound to the NAC core-binding site in *OsWRKY62* promoter and activated *OsWRKY62* expression (Figure 4). It is thus likely that *OsWRKY62* is a target of ONAC066 and ONAC066 modulates the expression of *OsWRKY62* through direct binding to NAC core-binding site in *OsWRKY62* promoter. Among these four WRKY genes up-regulated in *ONAC066*-OE plants, only *OsWRKY62* was identified as one of the ONAC066 targets (Figure 4). The portion between verified ONAC066 binding and up-regulated expression of the genes in *ONAC066*-OE plants was 25%, which is similar to values reported for other plant TFs such as Arabidopsis WRKY33 (Liu et al., 2015). The altered expression of *OsWRKY45* and *OsWRKY76* in *ONAC066*-OE and *ONAC066*-Ri plants may be affected indirectly by the ONAC066-activated defense signaling pathway. *OsWRKY45*, a central component of the SA signaling pathway, is a positive regulator of rice immunity (Shimono et al., 2007, 2012; Goto et al., 2015, 2016). However, inconsistent conclusions regarding the function of *OsWRKY62* in rice immunity were previously reported; for example, *OsWRKY62* and *OsWRKY76* are negative regulators of rice immunity against blast and bacterial leaf blight diseases (Peng et al., 2008, 2010; Liu et al., 2016; Liang et al., 2017) while *OsWRKY62* was reported to play a positive role in rice immunity (Fukushima et al., 2016). OsWRKY62 can form homodimers and heterodimers with OsWRKY45, and the OsWRKY45-OsWRKY62 heterodimer acts as a strong activator while the OsWRKY62 homodimer acts as a repressor of rice immunity (Fukushima et al., 2016). Simultaneous up-regulation of *OsWRKY45* and *OsWRKY62* in *ONAC066*-OE plants implies the formation of the OsWRKY45-OsWRKY62 heterodimers, which leads to the activation of defense response.

Due to limited number of up-regulated genes was identified in *ONAC066*-OE plants, we tried to mine putative target genes of ONAC066 through co-expression analysis, which has been successfully used to establish the transcriptional regulatory network and identify target genes for given TFs (Fang et al., 2015; Smita et al., 2015). Hundreds of functional genes were found to be co-expressed with *ONAC066* through two different online tools CREP and PLANEX (Supplementary Tables S4, S5). Among 15 co-expressed genes simultaneously identified in two different databases, 5 encode cytochrome P450s (Table 3). Cytochromes P450s constitute one of the largest families of enzymatic proteins with diverse functions from critical structural components to key signaling molecules (Mizutani and Ohta, 2010; Nelson and Werck-Reichhart, 2011; Pandian et al., 2020). Rice genome harbors 326 genes coding for 355 cytochrome P450s (Wei and Chen, 2018). Some of rice cytochrome P450s are involved in biosynthesis of diterpenoid phytoalexins and therefore play roles in resistance against fungal and bacterial pathogens (Wang Q. et al., 2011; Li et al., 2013, 2015; Maeda et al., 2019; Shen et al., 2019; Kitaoka et al., 2021; Liang et al., 2021). In our gene expression profiling and co-expression analysis, a set of cytochrome P450 genes were identified to be co-expressed with *ONAC066* (Table 3). The co-expression feature of 5 cytochrome P450 genes was confirmed by their up-regulated expressions in *ONAC066*-OE plants and down-regulated expressions in *ONAC066*-Ri plants (Figure 5). ONAC066 directly bound to the promoters of 3 co-expressed cytochrome P450 genes, *LOC_Os02g30110*, *LOC_Os06g37300* and *LOC_Os02g36150*, and activated their transcription (Figure 6), demonstrating that the *LOC_Os02g30110*, *LOC_Os06g37300*, and *LOC_Os02g36150* cytochrome P450 genes are ONAC066 targets. The fact that *DPF*, a positive regulator of biosynthesis of diterpenoid phytoalexins (Yamamura et al., 2015), was up-regulated in *ONAC066*-OE plants but down-regulated in *ONAC066*-Ri plants (Figure 5) imply that ONAC066 may play a role in regulating biosynthesis of diterpenoid phytoalexins in rice. Another, among the co-expressed cytochrome P450 genes, *OsCAld5H1* (LOC_Os10g36848) has been shown to be involved in regulating the structure of cell wall lignin (Takeda et al., 2017, 2019), which is related to rice immunity against *M. oryzae* and *X. oryzae* pv. *oryzae* (Liu et al., 2017; Li et al., 2020; Onohata and Gomi, 2020; Shamsunnaher et al., 2020). It is likely that ONAC066 contributes positively to rice immunity through direct regulation of a set of cytochrome P450s with distinct functions in biosynthesis of diterpenoid phytoalexins, cell wall lignin and other secondary metabolisms. However, the involvement of these co-expressed cytochrome P450s in rice immunity needs further investigation.

## Conclusion

In the present study, we further demonstrated that ONAC066 is a positive regulator of rice immunity against *M. oryzae* and identified *OsWRKY62* and three cytochrome P450 genes as ONAC066 targets. Therefore, ONAC066 contributes positively to rice immunity through regulating *OsWRKY62* expression to activate defense response and/or activating a set of cytochrome P450 genes to promote the biosynthesis of defense compounds including phytoalexins. Together with our previous study on the function of *ONAC066* in abiotic stress response (Yuan et al., 2019b), we concluded that *ONAC066* acts as a positive regulator of abiotic and biotic stress response in rice and thus can be used in creating novel rice germplasm with improved abiotic stress tolerance and disease resistance.

## Data Availability Statement

The original contributions presented in the study are included in the article/Supplementary Material, further inquiries can be directed to the corresponding author/s.

## Author Contributions

XY, DL, and FS conceived the project, designed the experiments, analyzed the data, and drafted the manuscript. XY generated all material used in this study (cloning, vector, transformations, and transgenic plants). XY, HW, YB, YY, YG, XX, and JW performed the experiments and collected the data. All authors commented on the manuscript.

## Conflict of Interest

The authors declare that the research was conducted in the absence of any commercial or financial relationships that could be construed as a potential conflict of interest.

## Publisher’s Note

All claims expressed in this article are solely those of the authors and do not necessarily represent those of their affiliated organizations, or those of the publisher, the editors and the reviewers. Any product that may be evaluated in this article, or claim that may be made by its manufacturer, is not guaranteed or endorsed by the publisher.

## Footnotes

1
http://bioinfo.cau.edu.cn/agriGO/analysis.php

2
http://crep.ncpgr.cn

3
http://planex.plantbioinformatics.org/

## Supplementary Material

Supplementary Figure S1 ONAC066 could not bind the promoters of OsWRKY45, OsWRKY76 and OsWRKY79.

Supplementary Figure S2 Expression patterns of identified co-expressed cytochrome P450 genes in *ONAC066*-OE and *ONAC066*-Ri plants.

Supplementary Table S1 List of primers used in this study.

Supplementary Table S2 Down-regulated genes in *ONAC066*-OE plants.

Supplementary Table S3 GO terms for up- and down-regulated genes in *ONAC066*-OE plants.

Supplementary Table S4 A 84 genes co-expressed with *ONAC066* from CREP database.

Supplementary Table S5 A 114 genes co-expressed with *ONAC066* from PALNEX database.
