# Supplementary material for: ONAC066, A Stress-Responsive NAC Transcription Activator, Positively Contributes to Rice Immunity Against Magnaprothe oryzae Through Modulating Expression of OsWRKY62 and Three Cytochrome P450 Genes
Source: Front Plant Sci. 2021 Sep 9;12:749186. doi: 10.3389/fpls.2021.749186 (PMC8458891; doi:10.3389/fpls.2021.749186)
Supplement: Supplementary Figure S1 — ONAC066 could not bind the promoters of OsWRKY45, OsWRKY76 and OsWRKY79. [file Data_Sheet_1.docx]

**Supplemental Table S1 |** List of primers used in this study.

| **Purpose** | **Primers** | **Prime sequence (5’-3’)** |
| --- | --- | --- |
| ***onac066-1***  **genotyping** | F1 | CAGGAGGTCTGGACCATCTG |
|  | R1 | CGTGTGCAGCTTTGAATGAC |
|  | R2/2707RB | CTGTGGAATTGATCAGCGTT |
| **Transient**  **expression**  **assay** | GAL4-DBD-F | GGACTAGTGATGAAGCTACTGTCTT |
|  | GAL4-DBD-R | CGTCTAGAGGCGATACAGTCAAC |
|  | GAL4-066-F | GGACTAGTGATGAAGCTACTGTCTT |
|  | GAL4-066-R | CGTCTAGACCTCAATATCTACAGTCAT |
|  | GAL4-066N-F | GGACTAGTGATGAAGCTACTGTCTT |
|  | GAL4-066N-R | GTCTAGACCGAGGCGGAAC |
|  | GAL4-066C-F | GCTCTAGAGTGCATGCAAGAAG |
|  | GAL4-066C-R | CGTCTAGACCTCAATATCTACAGTCAT |
|  | GAL4-022-F | GGACTAGTGATGAAGCTACTGTCTT |
|  | GAL4-022-R | CGTCTAGAGGTTAGTAGTAGAGCAGAT |
|  | LUC-pW62-F | GGAAGCTTAACACATAGCACCACACTCAT |
|  | LUC-pW62-R | GGGATCCGGCGGCAGCTAA |
|  | LUC-p02g30110-F | GGCTCGAGGATTGGTTAGGAAG |
|  | LUC-p02g30110-R | GGGGATCCTCATTATACCAGAAAGTA |
|  | LUC-p02g36150-F | GGCTCGAGCTTTTAAAGGGGTTG |
|  | LUC-p02g36150-R | GGGGATCCCTTAATTGATCGTCG |
|  | LUC-p06g37300-F | GGCTCGAGAAAATTCTTGACCGTG |
|  | LUC-p06g37300-R | GGGGATCCATGGCACACGATC |
| **qPCR** | q18s-F | ATGGTGGTGACGGGTGAC |
|  | q18s-R | CAGACACTAAAGCGCCCGGTA |
|  | q066-F | TGCATGCAAGAAGCTGAGGTCTG |
|  | q066-R | TGCTGCTTCCTGTAGGTGATGC |
|  | MoPot2-F | ACGACCCGTCTTTACTTATTTGG |
|  | MoPot2-R | AAGTAGCGTTGGTTTTGTTGGAT |
|  | OsUbi-F | TTCTGGTCCTTCCACTTTCAG |
|  | OsUbi-R | ACGATTGATTTAACCAGTCCATGA |
|  | qPR1-F | CCCTTTTTAATTAGCAATTAGAGAGCT |
|  | qPR1-R | CGAGCACTGCAGCTACTAGCAAGCTG |
|  | qPR2-F | CCTTCACCAAGTATCTGCGA |
|  | qPR2-R | GTCTAGCGCATTCTGCAAAC |
|  | qPR8-F | TCTACGACGTGCAGAACAACTTCAG |
|  | qPR8-R | TCCAACTCAACCACTGTGCAAGTAA |
|  | qPR1b-F | ACGGGCGTACGTACTGGCTA |
|  | qPR1b-R | CTCGGTATGGACCGTGAAG |
|  | qW45-F | TGTCACGTCCTCCCTGCACT |
|  | qW45-R | TCGTCCAGATCGAAGACAACC |
|  | qW62-F | ACCCCTACCCTAGAGCTTACTTCC |
|  | qW62-R | GACCTATCCTCCGCACATCTTT |
|  | qMYB-F | TTGGAAGCGCATAGAAGACCTC |
|  | qMYB-R | CTTGCAACTCGCAGATAAGAACG |
|  | q01g41820-F | GACAATGATCCTCTACGA |
|  | q01g41820-R | GTAAGTGATGCCTCCTAT |
|  | q02g30110-F | GGGATTACAATGCCAAAGA |
|  | q02g30110-R | TTAGGACGAAAGAAGATGAAC |
|  | q02g36150-F | GCCTAAGGACCTTGATAT |
|  | q02g36150-R | CTTGGAGCAATATGAGTAAT |
|  | q03g25480-F | CATAGCAACAATACTCAATC |
|  | q03g25480-R | AGGTAATTCTGGACTGTA |
|  | q06g37300-F | AAGGAGGCGATGATTGAC |
|  | q06g37300-R | TTACGAGTTAGCACAGACATT |
|  | q01g43851-F | TTCCATCTCGAGAAGCTGAAGAGG |
|  | q01g43851-R | TCTGCTTATCAGCTCGCTGGAAC |
|  | q01g52790-F | CTGAAAGAGCACGGGAAACTGTC |
|  | q01g52790-R | GGGTCTCTGATAATCACGAATGGC |
|  | q01g59000-F | AGCTGTTCTCGACGACTTCATTAG |
|  | q01g59000-R | CAGTAGAAGAACAGGCGAGAGC |
|  | q02g32770-F | ATCATCGTCGTGCTTCTGTTTGAC |
|  | q02g32770-R | TTGGCCACCGAATAAGCTCTGC |
|  | q02g36190-F | TGCGGTGTCCAAGAGTAATGGC |
|  | q02g36190-R | TGTGATCCTAGTCTTCCCAACAGC |
|  | q03g39690-F | TTGATCACTAACGTCGTCTTGGAC |
|  | q03g39690-R | TCTGTTAAGGCCCACTCAAGTGTC |
|  | q07g44130-F | TGGAGTGTTGAATGCAGCGAAG |
|  | q07g44130-R | TTTGCCCGATGCACGACCTTAG |
|  | q10g30410-F | TGGACTTGTTGGGCCTAAACGC |
|  | q10g30410-R | AAACACTGCTTGGAGCAACACG |
|  | q10g36848-F | AGAATGGTTTGCAGGTGATGGTG |
|  | q10g36848-R | AGCTAAGCTAGCCACACCAAGC |
| **Y1H** | Rec2-066-F | GAGTGGCCATTATGGcccATGGTGACCAGCAA |
|  | Rec2-066-R | GCCGACATGTTTTTTcccATATCTACAGTCATAGAATCC |
|  | HIS2-W62-F | GGAGCTCTTGGCAACAATGAGTTGTGG |
|  | HIS2-W62-R | GGACTAGTGGCGGCAGCTAAGCTAGC |
|  | HIS2-pW45-F | GGACTAGTCTCAACAGTGACAACTAGATCCA |
|  | HIS2-pW45-R | GGACTAGTCCTCAATCCAAGCAAGCA |
|  | HIS2-pW76-F | GGGAATTCGCTTGCACCGGTTCCTCT |
|  | HIS2-pW76-R | GGACTAGTCGACGACGACGACGACC |
|  | HIS2-pW79-F | GGGAGCTCGCACTCACTTGGTTCCGG |
|  | HIS2-pW79-R | GGACGCGTGCTATAGCTAGCTTAGCCAATTAC |
|  | HIS2-02g36150-R | GGACTAGTCTTAATTGATCGTCGGCG |
|  | HIS2-03g25480-F | GGGAATTCACGAAGACTACTTAATCCA |
|  | HIS2-03g25480-R | GGACTAGTGGCCGCAGCTAGC |
|  | HIS2-06g37300-F | GGGAATTCAAAATTCTTGACCGTG |
|  | HIS2-06g37300-R | GGACTAGTATGGCACACGATCTGATC |
| **ChIP-PCR** | P1-F | AGTCTAAATAGACTTGACCTTTTTG |
|  | P1-R | GCTTTCATAGAATTTTAATGAGATT |
|  | P2-F | CTCTAACATCCCACATACCGGA |
|  | P2-R | CATTGACATCGAGCTTACAACG |
|  | P3-F | GTAAACGTCAGTCTGTCATCTTTAA |
|  | P3-R | CTTCTACGGAATTAATGTTTTACC |
|  | P4-F | AGTAGAATTTTGATATCCTACGAGGC |
|  | P4-R | CGTGAACTTTGTGCGAGGATAA |
|  | P5-F | AATGTTTGACTTGACGCCG |
|  | P5-R | CATAACCCTCCACCATTCACT |
|  | P6-F | ATTGGTTAGGAAGAGAATGTTGG |
|  | P6-R | GCGGGCTGCTATGATTACAC |
|  | P7-F | CCCGCTGTTTCCATAACAA |
|  | P7-R | TGGCGGTGTCAAAATTTTG |
|  | P8-F | AATTTTGACACCGCCAAAAT |
|  | P8-R | AATGTGCATTCATTATACCAGAAA |
|  | P9-F | TTTATCGATACTATGCTATTAGCCA |
|  | P9-R | TCAGTTCTTTATCTAGTGTCGACC |
|  | P10-F | TAAAGAACTGACGTAAATTCTTTGAG |
|  | P10-R | CATGTAGTAGGTAGGTATTCATCGC |
|  | P11-F | CATGTGGCTCCCAAGGAGT |
|  | P11-R | CACACTAGAATAATAAGTCTATACGTACTATTC |
|  | P12-F | AGACTTATTATTCTAGTGTGTTTGAAAGA |
|  | P12-R | TGGCACACGATCTGATCACA |
|  | P13-F | CTCGGACAGCGCACGCTC |
|  | P13-R | TAAAAGGACCCCCGAGCTCTC |
|  | P14-F | GGGGTTGGAGGGCGACG |
|  | P14-R | TTTGGCTTTGGGCCGAAAG |
|  | P15-F | AAGAGACTTTTTAAAACTTTTTTCAAT |
|  | P15-R | CATTTCGAAACGGAGGGAG |
|  | P16-F | GAAATGTTTGACACCGTTAACTT |
|  | P16-R | CTTAATTGATCGTCGGCGT |

**Supplementary Table S2 |** Down-regulated genes in ONAC066-OE plants.

| TIGR ID | Description | Fold Change (OE11/WT) | q-value (%) (OE11/WT) |
| --- | --- | --- | --- |
| LOC_Os12g43450 | Thaumatin | 0.49 | 0.65 |
| LOC_Os02g10780 | SPX, N-terminal domain containing protein | 0.48 | 1.56 |
| LOC_Os07g27300 | LUC7 related family protein | 0.47 | 0.00 |
| LOC_Os06g22380 | Nucleic acid-binding, OB-fold domain protein | 0.46 | 0.65 |
| LOC_Os04g41680 | Similar to Chitinase | 0.46 | 0.65 |
| LOC_Os06g29730 | Conserved hypothetical protein | 0.45 | 0.00 |
| LOC_Os11g37970 | Similar to Chitin-binding allergen Brar2 | 0.45 | 4.39 |
| LOC_Os02g13660 | Proline-and threonine-rich protein | 0.44 | 2.89 |
| LOC_Os12g15530 | Cupredoxin domain containing protein | 0.41 | 0.65 |
| LOC_Os02g42370 | Protein kinase domain containing protein | 0.41 | 0.00 |
| LOC_Os10g05820 | Unknown function DUF1210 protein | 0.40 | 2.08 |
| LOC_Os05g12450 | UDP-glucuronosyl/UDP-glucosyltransferase | 0.40 | 3.07 |
| LOC_Os01g29804 | Conserved hypothetical protein | 0.38 | 2.36 |
| LOC_Os04g45834 | HAT dimerisation domain protein | 0.37 | 0.00 |
| LOC_Os02g53130 | Similar to Nitrate reductase | 0.37 | 0.00 |
| LOC_Os02g28170 | Transferase family protein | 0.35 | 0.00 |
| LOC_Os08g23870 | Late embryogenesis abundant (LEA) protein | 0.30 | 0.65 |
| LOC_Os01g23710 | Conserved hypothetical protein | 0.29 | 0.00 |
| LOC_Os12g21784 | Similar to Protein kinase | 0.27 | 0.00 |
| LOC_Os12g19304 | Hypothetical protein | 0.25 | 0.65 |
| LOC_Os11g22350 | ABC transporter related protein | 0.13 | 0.00 |
| Os05g0563300 | Conserved hypothetical protein | 0.48 | 3.07 |
| Os08g0285301 | Conserved hypothetical protein | 0.44 | 1.13 |
| Os01g0879800 | Conserved hypothetical protein | 0.41 | 0.65 |
| Os.39000.1.S1_at |  | 0.49 | 0.65 |
| Os.16037.1.S1_at |  | 0.45 | 3.07 |

Averages of fold changes from three independent biological samples were shown.

q-value (%) shows the significance analysis based on three independent biological replicates. Genes with a cutoff q-value< 5% and fold changes <0.5 were defined as down-regulated expressed genes.

**Supplementary Table 3 |** GO terms for up- and down-regulated genes in ONAC066-OE plants.

| GO term | Description | Number in  input list ^a^ | Number in  background ^b^ | *p*-value | FDR ^c^ |
| --- | --- | --- | --- | --- | --- |
| ***Up-regulated*** | | | | | |
| GO:0044238 | primary metabolic process | 17 | 6775 | 0.0022 | 0.056 |
| GO:0008152 | metabolic process | 19 | 7746 | 0.0015 | 0.056 |
| GO:0043687 | post-translational protein modification | 6 | 1236 | 0.0039 | 0.06 |
| GO:0009987 | cellular process | 18 | 8160 | 0.0066 | 0.06 |
| GO:0006464 | protein modification process | 6 | 1359 | 0.0062 | 0.06 |
| GO:0043412 | macromolecule modification | 6 | 1406 | 0.0073 | 0.06 |
| GO:0043170 | macromolecule metabolic process | 13 | 5520 | 0.014 | 0.099 |
| GO:0005488 | binding | 24 | 8681 | 0.000032 | 0.0023 |
| GO:0003824 | catalytic activity | 21 | 8329 | 0.00049 | 0.018 |
| GO:0003677 | DNA binding | 7 | 1445 | 0.0019 | 0.024 |
| GO:0001883 | purine nucleoside binding | 6 | 1171 | 0.003 | 0.024 |
| GO:0016491 | oxidoreductase activity | 6 | 1141 | 0.0027 | 0.024 |
| GO:0001882 | nucleoside binding | 6 | 1171 | 0.003 | 0.024 |
| GO:0005524 | ATP binding | 6 | 1071 | 0.0019 | 0.024 |
| GO:0030554 | adenyl nucleotide binding | 6 | 1171 | 0.003 | 0.024 |
| GO:0032559 | adenyl ribonucleotide binding | 6 | 1074 | 0.002 | 0.024 |
| GO:0032555 | purine ribonucleotide binding | 6 | 1218 | 0.0037 | 0.024 |
| GO:0032553 | ribonucleotide binding | 6 | 1218 | 0.0037 | 0.024 |
| GO:0017076 | purine nucleotide binding | 6 | 1317 | 0.0053 | 0.032 |
| GO:0043169 | cation binding | 8 | 2582 | 0.013 | 0.066 |
| GO:0043167 | ion binding | 8 | 2584 | 0.013 | 0.066 |
| GO:0000166 | nucleotide binding | 6 | 1686 | 0.017 | 0.08 |
| GO:0005623 | cell | 48 | 30433 | 0.0007 | 0.02 |
| GO:0044464 | cell part | 48 | 30433 | 0.0007 | 0.02 |
| GO:0031982 | vesicle | 17 | 7454 | 0.0061 | 0.058 |
| GO:0016023 | cytoplasmic membrane-bounded vesicle | 17 | 7445 | 0.0061 | 0.058 |
| GO:0031410 | cytoplasmic vesicle | 17 | 7454 | 0.0061 | 0.058 |
| GO:0005634 | nucleus | 7 | 1842 | 0.007 | 0.058 |
| GO:0031988 | membrane-bounded vesicle | 17 | 7445 | 0.0061 | 0.058 |
| ***Down-regulated*** | | | | | |
| GO:0016023 | cytoplasmic membrane-bounded vesicle | [9](http://bioinfo.cau.edu.cn/agriGO/termDetail.php?session=901027103&GO=GO:0031982) | 7445 | 0.0011 | 0.015 |
| GO:0031988 | membrane-bounded vesicle | [9](http://bioinfo.cau.edu.cn/agriGO/termDetail.php?session=901027103&GO=GO:0016023) | 7445 | 0.0011 | 0.015 |
| GO:0031982 | vesicle | [9](http://bioinfo.cau.edu.cn/agriGO/termDetail.php?session=901027103&GO=GO:0031410) | 7454 | 0.0011 | 0.015 |
| GO:0031410 | cytoplasmic vesicle | [9](http://bioinfo.cau.edu.cn/agriGO/termDetail.php?session=901027103&GO=GO:0031988) | 7454 | 0.0011 | 0.015 |

^a^, Significant over-represented GO categories were determined by AgriGO SEA using a Fisher test and a Bonferroni multi-test correction.

^b^, *Oryza sativa* subsp. *japonica* Affymetrix Genome Array GPL2025, which consists of 45078 annotations, was chosen as background.

^c^, FDR, false discovery rate.

**Supplementary Table 4 |** 84 genes co-expressed with *ONAC066* from CREP database.

| **TIGR locus ID** | **Description** | **PCC** |
| --- | --- | --- |
| LOC_Os11g06234 | Expressed protein | 0.95 |
| **LOC_Os01g18584** | **OsWRKY9** | **0.91** |
| **LOC_Os02g19550** | **Lectin receptor-type protein kinase** | **0.90** |
| **LOC_Os01g70210** | **Phosphatidylinositol transfer protein CSR1** | **0.89** |
| LOC_Os03g20330 | VQ motif family protein | 0.89 |
| **LOC_Os07g38800** | **Lectin-like receptor kinase 7** | **0.88** |
| **LOC_Os07g44130** | **Cytochrome P450 72A1** | **0.87** |
| **LOC_Os06g07200** | **Syntaxin 132** | **0.86** |
| **LOC_Os07g38810** | **Lectin receptor-type protein kinase** | **0.85** |
| LOC_Os07g03040 | Expressed protein | 0.85 |
| LOC_Os07g48800 | VQ motif family protein | 0.85 |
| LOC_Os12g24650 | Leucine aminopeptidase 1 | 0.85 |
| LOC_Os07g33780 | PDR-like ABC transporter | 0.84 |
| LOC_Os10g04490 | Conserved hypothetical protein | 0.84 |
| **LOC_Os01g41820** | **Cytochrome P450 72A1** | **0.84** |
| LOC_Os11g10120 | Expressed protein | 0.83 |
| **LOC_Os06g18820** | **Expressed protein** | **0.83** |
| LOC_Os01g60120 | Transmembrane protein 56 | 0.83 |
| LOC_Os01g37750 | Glutathione S-transferase GSTU6 | 0.82 |
| LOC_Os09g04310 | Expressed protein | 0.82 |
| LOC_Os12g43750 | Expressed protein | 0.82 |
| LOC_Os06g29844 | Transparent testa 12 protein | 0.82 |
| LOC_Os06g46160 | Expressed protein | 0.81 |
| LOC_Os10g17960 | Receptor-like protein kinase homolog RK20-1 | 0.81 |
| LOC_Os10g40490 | U-box domain containing protein | 0.81 |
| LOC_Os11g15520 | Peptidase subtilase | 0.80 |
| LOC_Os03g10100 | Sorbitol transporter | 0.80 |
| **LOC_Os04g15580** | **Serine threonine-protein kinase receptor** | **0.80** |
| LOC_Os01g49614 | Ser Thr protein kinase | 0.80 |
| LOC_Os10g05250 | Protein kinase domain containing protein | 0.80 |
| LOC_Os10g36848 | Cytochrome P450 84A1 | 0.80 |
| LOC_Os12g10660 | Salt tolerance-like protein | 0.79 |
| LOC_Os03g39690 | Cytochrome P450 71D7 | 0.79 |
| LOC_Os07g45570 | Expressed protein | 0.79 |
| LOC_Os05g43390 | Signal recognition particle 54 kDa protein | 0.79 |
| **LOC_Os01g59000** | **Cytochrome P450 94A2** | **0.79** |
| LOC_Os11g07930 | Retinol dehydrogenase 13 | 0.78 |
| LOC_Os10g30410 | Cytochrome P450 71D7 | 0.78 |
| LOC_Os12g43970 | Epoxide hydrolase 2 | 0.78 |
| LOC_Os01g06920 | Resistance protein SlVe1 precursor | 0.78 |
| LOC_Os04g37490 | NAD(P)H-dependent oxidoreductase | 0.78 |
| LOC_Os04g47770 | Cis-zeatin O-glucosyltransferase | 0.78 |
| LOC_Os09g09500 | Lectin-like receptor kinase 7 | 0.78 |
| LOC_Os04g38540 | Aldose 1-epimerase | 0.78 |
| LOC_Os01g58280 | Cucumisin precursor | 0.78 |
| LOC_Os01g02840 | Resistance-related receptor-like kinase | 0.78 |
| LOC_Os09g09510 | Protein kinase | 0.78 |
| LOC_Os03g03730 | Regulatory protein | 0.78 |
| LOC_Os02g39884 | Expressed protein | 0.78 |
| **LOC_Os02g30110** | **Cytochrome P450** | **0.78** |
| LOC_Os07g03170 | Expressed protein | 0.77 |
| LOC_Os09g13440 | Expressed protein | 0.77 |
| LOC_Os11g31530 | BIN1-associated receptor kinase | 0.77 |
| LOC_Os10g21630 | Expressed protein | 0.77 |
| LOC_Os07g34980 | Protein kinase domain containing protein | 0.77 |
| LOC_Os04g44300 | Transposon protein | 0.77 |
| LOC_Os03g56160 | Lectin-like receptor kinase 7 | 0.77 |
| LOC_Os11g11970 | Expressed protein | 0.77 |
| LOC_Os01g43851 | Cytochrome P450 72A1 | 0.77 |
| LOC_Os01g02810 | Receptor kinase LRK14 | 0.77 |
| LOC_Os07g07300 | Expressed protein | 0.77 |
| LOC_Os03g24930 | Protein kinase APK1A | 0.77 |
| **LOC_Os01g14590** | **Pathogen-related protein** | **0.76** |
| LOC_Os08g25430 | Cysteine-rich receptor-like protein kinase | 0.76 |
| LOC_Os06g06350 | ACS-like protein | 0.76 |
| LOC_Os07g03050 | Expressed protein | 0.76 |
| LOC_Os09g26144 | Glutamate receptor 2.8 precursor | 0.76 |
| LOC_Os01g53390 | Anthocyanidin 5,3-O-glucosyltransferase | 0.76 |
| LOC_Os11g29870 | OsWRKY72 | 0.76 |
| **LOC_Os01g52790** | **Cytochrome P450 72A1** | **0.76** |
| **LOC_Os12g25170** | **Disease resistance protein RGA2** | **0.76** |
| LOC_Os02g32770 | Cytochrome P450 71D6 | 0.76 |
| LOC_Os10g04520 | Expressed protein | 0.75 |
| LOC_Os10g39710 | Strictosidine synthase | 0.75 |
| LOC_Os03g10450 | Sua5/YciO/YrdC/YwlC family protein | 0.75 |
| LOC_Os06g51070 | NAC domain-containing protein 68 | 0.75 |
| LOC_Os06g41100 | TGA10 transcription factor | 0.75 |
| LOC_Os01g64440 | Expressed protein | 0.75 |
| LOC_Os09g39620 | ATP binding protein | 0.75 |
| LOC_Os03g61829 | Haloacid dehalogenase-like hydrolase | 0.75 |
| LOC_Os01g63180 | L-ascorbate oxidase precursor | 0.75 |
| LOC_Os03g25480 | Cytochrome P450 72A1 | 0.75 |
| LOC_Os10g07978 | Disease resistance protein RPM1 | 0.75 |
| LOC_Os04g24220 | OsWAK32 | 0.75 |

The bolded genes were found in both CREP and PLAEX databases.

**Supplementary Table 5 |** 114 genes co-expressed with *ONAC066* from PALNEX database.

| **TIGR locus ID** | **Description** | **PCC** |
| --- | --- | --- |
| LOC_Os03g20330 | VQ protein, expressed | 0.70 |
| **LOC_Os07g38810** | **Receptor lectin kinase** | **0.68** |
| LOC_Os07g35690 | Cysteine-rich RLK 10 | 0.68 |
| LOC_Os04g56110 | Protein kinase, putative, expressed | 0.66 |
| LOC_Os06g51170 | Protein kinase superfamily protein | 0.65 |
| **LOC_Os12g25170** | **LRR and NB-ARC protein** | **0.65** |
| **LOC_Os07g38800** | **Receptor lectin kinase** | **0.64** |
| LOC_Os06g37300 | Cytochrome P450 | 0.63 |
| **LOC_Os06g07200** | **Syntaxin of plants 131** | **0.63** |
| LOC_Os02g36190 | Cytochrome P450 | 0.62 |
| LOC_Os07g18230 | Receptor lectin kinase | 0.62 |
| LOC_Os06g45890 | Homeodomain-like superfamily protein | 0.62 |
| LOC_Os01g70130 | Protein kinase superfamily protein | 0.62 |
| LOC_Os10g40490 | U-box domain protein | 0.62 |
| LOC_Os02g36210 | Terpenoid cyclases | 0.62 |
| **LOC_Os01g52790** | **Cytochrome P450** | **0.62** |
| LOC_Os03g18560 | Protein of unknown function DUF538 | 0.61 |
| LOC_Os04g54020 | Protein serine/threonine kinases | 0.61 |
| LOC_Os10g42040 | Expressed protein | 0.61 |
| LOC_Os09g24924 | Sugar transporter protein 7 | 0.61 |
| LOC_Os06g18000 | Leucine-rich repeat protein kinase | 0.61 |
| LOC_Os09g39940 | Uclacyanin 1 | 0.61 |
| LOC_Os05g45410 | Heat shock transcription factor A4A | 0.60 |
| **LOC_Os07g44130** | **Cytochrome P450** | **0.60** |
| LOC_Os06g50220 | Protein of unknown function (DUF668) | 0.60 |
| LOC_Os07g18990 | Lipid-transfer protein | 0.60 |
| LOC_Os08g05480 | Phloem protein 2-B2 | 0.60 |
| LOC_Os03g05470 | Calmodulin-binding receptor-like kinase | 0.59 |
| LOC_Os04g41310 | Protein kinase superfamily protein | 0.59 |
| **LOC_Os02g19550** | **Concanavalin A-like lectin protein kinase** | **0.59** |
| LOC_Os09g20480 | Major facilitator superfamily protein | 0.59 |
| LOC_Os11g09350 | VHS and GAT domain protein | 0.59 |
| LOC_Os07g46846 | NAD(P)-binding Rossmann-fold protein | 0.59 |
| LOC_Os03g38590 | Secretory carrier 3 | 0.59 |
| LOC_Os05g09020 | WRKY67 | 0.59 |
| LOC_Os06g44170 | NAD(P)-binding Rossmann-fold protein | 0.59 |
| LOC_Os02g05960 | Leucine-rich receptor-like protein kinase | 0.59 |
| LOC_Os06g45570 | VQ motif-containing protein | 0.59 |
| LOC_Os01g11520 | RING/U-box superfamily protein | 0.58 |
| LOC_Os07g18240 | Concanavalin A-like lectin protein kinase | 0.58 |
| LOC_Os11g10470 | Expressed protein | 0.58 |
| LOC_Os11g05490 | Expressed protein | 0.58 |
| LOC_Os09g39650 | U-box domain-containing protein kinase | 0.58 |
| LOC_Os01g50100 | P-glycoprotein 11 | 0.58 |
| LOC_Os01g64540 | UDP-Glycosyltransferase | 0.58 |
| LOC_Os10g39700 | Chitinase | 0.58 |
| LOC_Os03g61060 | Calmodulin-binding receptor-like kinase | 0.58 |
| **LOC_Os06g18820** | **Adenine nucleotide alpha hydrolases-like protein** | **0.58** |
| LOC_Os01g53920 | Protein kinase family protein | 0.58 |
| LOC_Os08g03350 | Lysine histidine transporter 1 | 0.58 |
| LOC_Os02g43430 | Protein kinase superfamily protein | 0.58 |
| LOC_Os02g04640 | Myb-like HTH transcriptional regulator | 0.58 |
| LOC_Os04g53810 | NAD(P)-binding Rossmann-fold protein | 0.58 |
| LOC_Os03g03700 | MLO domain containing protein | 0.57 |
| **LOC_Os01g14590** | **Pathogenesis-related protein** | **0.57** |
| **LOC_Os01g41820** | **Cytochrome P450** | **0.57** |
| LOC_Os10g25010 | OsCML8 | 0.57 |
| LOC_Os01g70490 | High affinity K+ transporter 5 | 0.57 |
| LOC_Os02g12090 | Expressed protein | 0.57 |
| **LOC_Os04g15580** | **Serine/threonine-protein kinase receptor** | **0.57** |
| LOC_Os02g07690 | VQ motif-containing protein | 0.57 |
| LOC_Os09g21180 | Homeobox associated leucine zipper | 0.57 |
| LOC_Os09g39960 | DYNAMIN-like 1E | 0.57 |
| LOC_Os01g02600 | PR5-like receptor kinase | 0.57 |
| **LOC_Os01g59000** | **Cytochrome P450** | **0.57** |
| LOC_Os04g49748 | Purine permease 11 | 0.56 |
| LOC_Os05g39240 | Ammonium transporter 2 | 0.56 |
| **LOC_Os02g30110** | **Cytochrome P450** | **0.56** |
| LOC_Os10g33140 | LRR family protein | 0.56 |
| LOC_Os03g04060 | Basic chitinase | 0.56 |
| LOC_Os07g03860 | Concanavalin A-like lectin protein kinase | 0.56 |
| LOC_Os02g36150 | Cytochrome P450 | 0.56 |
| LOC_Os09g08130 | Aldolase-type TIM barrel family protein | 0.56 |
| LOC_Os06g29730 | RALFL28 | 0.56 |
| LOC_Os05g51590 | Development and Cell Death protein | 0.56 |
| LOC_Os03g43770 | RNI-like superfamily protein | 0.56 |
| LOC_Os01g54350 | Protein kinase superfamily protein | 0.56 |
| LOC_Os10g34930 | Plant basic secretory protein family protein | 0.56 |
| LOC_Os05g05040 | Expressed protein | 0.56 |
| LOC_Os12g36880 | Pathogenesis-related Bet v I | 0.56 |
| LOC_Os07g42310 | Lung seven transmembrane receptor protein | 0.56 |
| LOC_Os08g41470 | Proteophosphoglycan ppg4 | 0.56 |
| LOC_Os04g31030 | RPM1 interacting protein 4 | 0.56 |
| **LOC_Os01g70210** | **Sec14p-like phosphatidylinositol transfer protein** | **0.56** |
| LOC_Os02g36285 | Terpenoid cyclases | 0.56 |
| LOC_Os08g39850 | lipoxygenase 2 | 0.56 |
| **LOC_Os01g18584** | **WRKY9** | **0.56** |
| LOC_Os02g14480 | Leucine-rich repeat protein kinase | 0.56 |
| LOC_Os07g33780 | Pleiotropic drug resistance 11 | 0.56 |
| LOC_Os12g05690 | Expressed protein | 0.55 |
| LOC_Os12g36850 | Pathogenesis-related Bet v I | 0.55 |
| LOC_Os10g04540 | LRR and NB-ARC protein | 0.55 |
| LOC_Os02g05920 | Leucine-rich receptor-like protein kinase | 0.55 |
| LOC_Os07g39350 | Polyol/monosaccharide transporter 5 | 0.55 |
| LOC_Os05g50100 | Expressed protein | 0.55 |
| LOC_Os07g03040 | Expressed protein | 0.55 |
| LOC_Os10g39010 | Protein kinase superfamily protein | 0.55 |
| LOC_Os01g53810 | Peptidase M28 family protein | 0.55 |
| LOC_Os05g41460 | Cysteine proteinase inhibitor | 0.55 |
| LOC_Os01g06280 | Hercules receptor kinase 1 | 0.55 |
| LOC_Os03g59180 | Expressed protein | 0.55 |
| LOC_Os11g05530 | Expressed protein | 0.55 |
| LOC_Os01g04409 | Protein kinase superfamily protein | 0.55 |
| LOC_Os01g69050 | Protein of unknown function (DUF707) | 0.55 |
| LOC_Os02g27310 | Cysteine-rich RLK 8 | 0.55 |
| LOC_Os06g03580 | RING/U-box superfamily protein | 0.55 |
| LOC_Os04g30930 | NB-ARC domain protein | 0.55 |
| LOC_Os09g26460 | RING/U-box superfamily protein | 0.55 |
| LOC_Os12g41710 | Protein kinase superfamily protein | 0.55 |
| LOC_Os09g28160 | phosphate transporter 3;2 | 0.55 |
| LOC_Os09g14490 | NB-ARC domain protein | 0.55 |
| LOC_Os12g29950 | Major facilitator superfamily protein | 0.55 |
| LOC_Os09g24620 | expressed protein | 0.55 |
| LOC_Os07g03840 | Concanavalin A-like lectin protein kinase | 0.55 |

The bolded genes were found in both CREP and PLAEX databases.


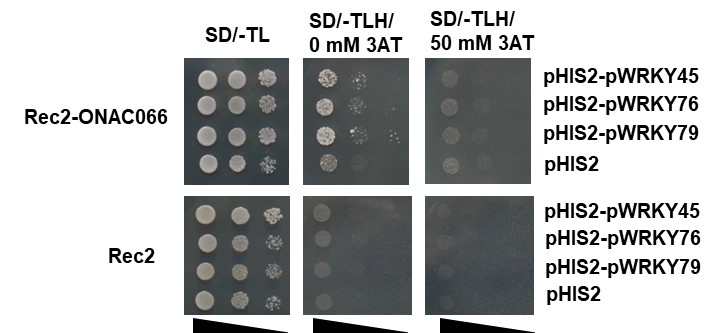


**Supplementary Figure 1 |** ONAC066 could not bind to the promoters of *OsWRKY45*, *OsWRKY76* and *OsWRKY79*. Growth performance of yeast transformants harboring expression construct Rec2-*ONAC066* and reporter construct *pHis2-pWRKY45/76/79* on SD/-Leu-/Trp/-His medium containing 50 mM 3-AT. Experiments were repeated at least three times with similar results.


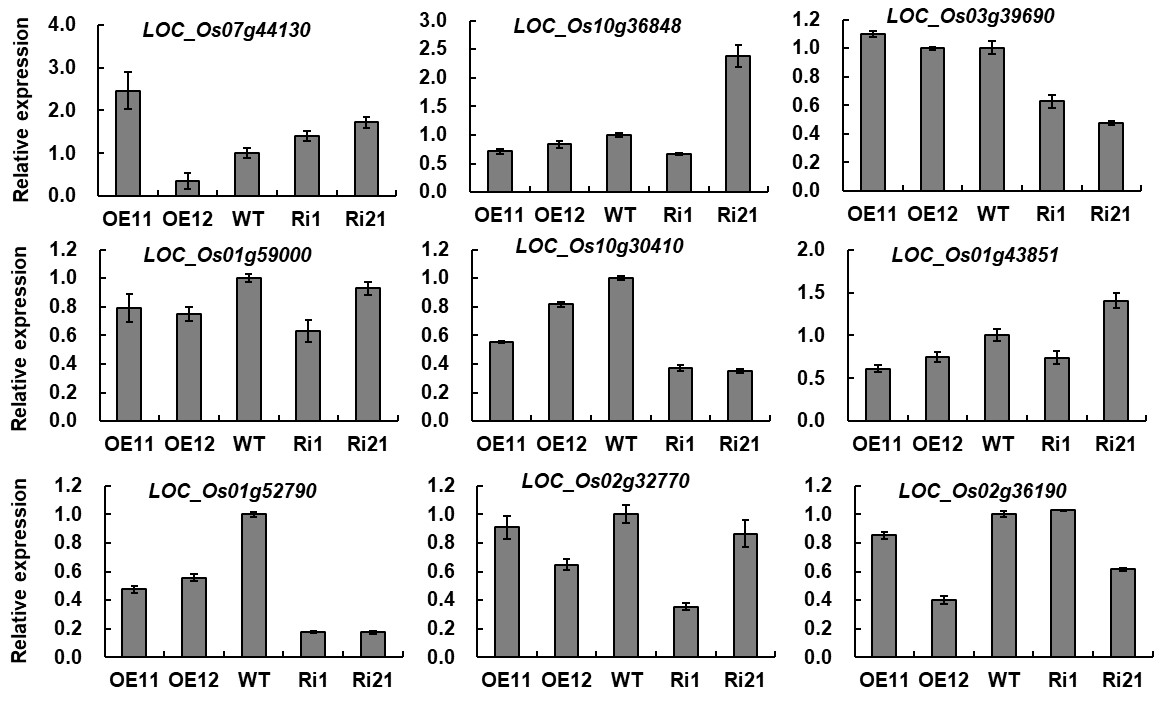


**Supplementary Figure 2 |** Expression patterns of identified co-expressed cytochrome P450 genes in ONAC066-OE and ONAC066-Ri plants. Data represented are the means ± SD from three independent experiments and no significant difference was detected between ONAC066-OE/ONAC066-Ri and WT plants.
